# Supplementary material for: Organising polypharmacy: unpacking medicines, unpacking meanings—an ethnographic study
Source: BMJ Open. 2021 Aug 26;11(8):e049218. doi: 10.1136/bmjopen-2021-049218 (PMC8395269; doi:10.1136/bmjopen-2021-049218)
Supplement: Author's manuscript [file bmjopen-2021-049218.draft_revisions.pdf]

BMJ Open

BMJ Open is committed to open peer review. As part of this commitment we make the peer review history of every article we publish publicly available.

When an article is published we post the peer reviewers' comments and the authors' responses online. We also post the versions of the paper that were used during peer review. These are the versions that the peer review comments apply to.

The versions of the paper that follow are the versions that were submitted during the peer review process. They are not the versions of record or the final published versions. They should not be cited or distributed as the published version of this manuscript.

BMJ Open is an open access journal and the full, final, typeset and author-corrected version of record of the manuscript is available on our site with no access controls, subscription charges or pay-per-view fees (http://bmjopen.bmj.com).

If you have any questions on BMJ Open's open peer review process please email info.bmjopen@bmj.com

BMJ Open

Organising polypharmacy: unpacking medicines, unpacking meanings – an ethnographic study

Journal:	*BMJ Open*
Manuscript ID	bmjopen-2021-049218
Article Type:	Original research
Date Submitted by the Author:	11-Feb-2021
Complete List of Authors:	Swinglehurst, Deborah; Queen Mary University of London Barts and The London School of Medicine and Dentistry, Institute of Population Health Sciences<br>Fudge, Nina ; Queen Mary University of London, Institute of Population Health Sciences
Keywords:	QUALITATIVE RESEARCH, PRIMARY CARE, THERAPEUTICS

SCHOLARONE™
Manuscripts

I, the Submitting Author has the right to grant and does grant on behalf of all authors of the Work (as defined in the below author licence), an exclusive licence and/or a non-exclusive licence for contributions from authors who are: i) UK Crown employees; ii) where BMJ has agreed a CC-BY licence shall apply, and/or iii) in accordance with the terms applicable for US Federal Government officers or employees acting as part of their official duties; on a worldwide, perpetual, irrevocable, royalty-free basis to BMJ Publishing Group Ltd ("BMJ") its licensees and where the relevant Journal is co-owned by BMJ to the co-owners of the Journal, to publish the Work in this journal and any other BMJ products and to exploit all rights, as set out in our licence.

The Submitting Author accepts and understands that any supply made under these terms is made by BMJ to the Submitting Author unless you are acting as an employee on behalf of your employer or a postgraduate student of an affiliated institution which is paying any applicable article publishing charge ("APC") for Open Access articles. Where the Submitting Author wishes to make the Work available on an Open Access basis (and intends to pay the relevant APC), the terms of reuse of such Open Access shall be governed by a Creative Commons licence – details of these licences and which Creative Commons licence will apply to this Work are set out in our licence referred to above.

Other than as permitted in any relevant BMJ Author's Self Archiving Policies, I confirm this Work has not been accepted for publication elsewhere, is not being considered for publication elsewhere and does not duplicate material already published. I confirm all authors consent to publication of this Work and authorise the granting of this licence.

For peer review only

Title: Organising polypharmacy: unpacking medicines, unpacking meanings – an ethnographic study

Authors

Deborah Swinglehurst MBBS, MA, MSc, PhD, FHEA, FRCGP
Institute of Population Health Sciences
Barts and The London School of Medicine and Dentistry
Queen Mary University of London
London, UK

Nina Fudge BA (Hons), MSc, PhD, FHEA
Institute of Population Health Sciences
Barts and The London School of Medicine and Dentistry
Queen Mary University of London
London, UK

Corresponding author:

Deborah Swinglehurst
Institute of Population Health Sciences
Barts and The London School of Medicine and Dentistry
Queen Mary University of London
58 Turner Street
London E1 2AB
UK

Email: d.swinglehurst@qmul.ac.uk

Word count: 3743 (excluding title page, abstract, article summary, references, and tables)

For peer review only - http://bmjopen.bmj.com/site/about/guidelines.xhtml

ABSTRACT

Objectives: We explore how older patients affected by polypharmacy manage the 'hidden work' of organising their medicines, how they make sense of this work and integrate it into their lives.

Design and setting: Ethnographic study observing patients over 18-24 months in patients' homes, general practice and community pharmacy, in England, UK.

Participants and methods: Ethnographic case study employing mixed qualitative methods including longitudinal follow-up of 24 patients aged 65 or older and prescribed ten or more items of medication. Our dataset includes: 562 hours of ethnographic observation across patients homes, community pharmacies and general practices; 47 audio-recorded interviews with patients about their lives and medicines practices; cultural probes (photographs, body maps, diaries and imagined 'wishful thinking' conversations); fieldnotes from regular home visits; telephone calls, and observation/video-recording of healthcare encounters. We apply a 'practice theory' lens to our analysis, illuminating what is being accomplished, why and by whom.

Results: All participants had developed strategies and routines for organising medicines into their lives, negotiating medicine-taking to enable acceptable adherence and make their medicines manageable. Strategies often involved the use of 'do-it-yourself' dosette boxes, and participants adopted a range of approaches to manage supplies and flex their regimens to align with personal values and priorities. Practices of organising medicines are effortful, creative and often highly collaborative. Patients strive for adherence, but their organisational efforts privilege 'living with medicines' over taking medicines strictly 'as prescribed'.

Conclusions

Polypharmacy demands careful organising. The burden of organising polypharmacy always falls somewhere, whether this is undertaken by pharmacists as they prepare multi-compartment compliance aids (MCCAs) or by patients in their homes. Greater appreciation amongst prescribers of the nature and complexity of this work may provide a useful point of departure for tackling the key issue that sustains it: polypharmacy.

For peer review only - http://bmjopen.bmj.com/site/about/guidelines.xhtml

Strengths and limitations of this study

- Gathers rich qualitative data from a range of community settings and is grounded in the everyday experiences of participants
- Reveals decision-making and practices around medication management which are often hidden from view and unknown to professionals who prescribe and dispense medicines
- Uses innovative qualitative methods to explore aspects of everyday life which might be difficult to reach using more conventional approaches
- Prioritises depth of analysis over breadth, enabling richness of conceptual insights but limiting generalisability

Keywords

Polypharmacy; Multimorbidity; Qualitative Research; Primary Health Care

For peer review only

INTRODUCTION

Polypharmacy is one of three key action areas in the World Health Organization Global Challenge 'Medication without Harm'.[1] Patients who are prescribed ten or more items of medication are at particular risk of harm.[2-4] Taking medication reliably and safely is demanding for patients, especially when complex medication regimens are involved.[5 6] Supporting patients in taking their medication is a key policy and professional commitment, enshrined within 'medicines optimisation': a "*person-centred approach to safe and effective medicines use, to ensure people obtain the best possible outcomes from their medicines*'.[4 7 8] Medicines optimisation is situated within an objective 'rational use of drugs' paradigm, which emphasises judicious prescribing by well-informed professionals of well-studied drugs to well-informed patients for well-defined conditions.[9] Achieving medicines optimisation is assumed to hinge centrally on a process of 'shared decision making' within the clinical consultation. This is emphasised in the NHS England 2020/21 update to the GP contract which requires Primary Care Networks to invite patients who are prescribed ten or more medications for a Structured Medication Review (SMR) with an appropriately trained clinical pharmacist or general practitioner.[10]

Notwithstanding the importance of medication reviews, most decision-making about medicines taking, and almost all of patients' medication practices,[11] take place beyond the clinic, usually in patients' homes. In the clinic, medicines are typically conceptualised as biomedical technologies (a disease, or risk of disease, is identified and a medicine is prescribed to ameliorate symptoms or reduce risk). But medicines can also be conceptualised as socially embedded phenomena, as representations that carry meanings and shape social relations.[12] Medicines are material *things* of therapy which have a social life,[13] and this becomes especially salient when medicines enter patients' homes and daily lives. Here, patients construct a 'lay pharmacology' of personally relevant notions of safety, side effects and efficacy which both converge with, and diverge from, conventional biomedical perspectives.[14] Medication practices become sites of moral evaluations of self, society and pharmaceuticals; peoples' relationship with medicines offers insight into their identities, and into these moral stories of self and society.[15] Consumption of medicines becomes a matter of embodied, purposeful, situated accomplishment,[6 16] involving a particular kind of *work* or expertise as patients devise ways of

For peer review only - http://bmjopen.bmj.com/site/about/guidelines.xhtml

incorporating their medicines into their daily lives. Patients have to ‘do’ medicines adherence.[6]

Under this view, taking medicines is not only a matter of cognition and decision-making (shared or otherwise). It is also a very practical matter of *organising*, of managing medicines *into* daily life and establishing routines. Indeed, one of the key motivations for establishing routines is to *minimise* the cognitive demands involved in carrying them through. A balance must be struck. As medicines routines become more reliable and the attention invested in them reduces, the unintentional risk of missing doses increases and further strategies must be devised to anticipate this possibility.[17 18] Huyard et al. studied unintentional non-adherence amongst patients prescribed single items of long term medication and found that patients used a range of strategies to support their adherence.[18] These included *anchoring* (scheduling pill-taking with another pre-existing routine), *spatial positioning* of pills in ways that make pill-taking more reliable, and methods for *verifying* that pill-taking has occurred.[18]

Polypharmacy, and in particular so-called ‘high risk polypharmacy’ involving ten or more separate items of medication,[4] is likely to present particular organisational challenges to both professionals and patients. If a genuinely *person-centred* approach to medicines optimisation is sought, research is needed which illuminates these challenges, by attending to professionals’ and patients’ subjective, context-specific evaluations of medicines, their lived experiences and everyday practices. When patients devise routines to support their medicines-taking they are invested in biographical work, in what Huyard describes as the ‘*microintegration of their illness deep into patients’ everyday lives*’.[18] Our research adopts an ethnographic approach to explore these practices.

In this paper, we focus on older adults who live at home and are prescribed ten or more separate items of medication. We begin with a brief ethnographic description of practices we observed in community pharmacy as professionals engaged in the preparation of multi-compartment compliance aids (MCCAs) or ‘dosette’ boxes, for some patients affected by polypharmacy. This draws attention to the organisation of polypharmacy as a form of ‘work’. We then shift our ethnographic gaze from the professional context into the private context of patients’ homes to address the question: ‘*How do patients organise their medicines and medicine-taking?*’ We

For peer review only - http://bmjopen.bmj.com/site/about/guidelines.xhtml

explore the meanings embedded in these everyday acts of organising and illuminate who is doing the work and what this work involves. We show how patients draw on personal, social, material and hermeneutical resources in their efforts to adhere to their medicines within the context of their daily lives, and how their lives are shaped by, and in turn shape, their medicines regimens.

METHODS

The study reported here is part of the wider APOLLO-MM study (Addressing the Polypharmacy Challenge in Older People with Multimorbidity) funded by the National Institute of Health Research (see www.polypharmacy.org.uk). The methods have been described in detail elsewhere;[19] those relevant to this paper are summarised below.

Setting and data collection

We recruited a purposive sample of 24 patients, 14 from two inner city general practices in an area of high deprivation, 10 from a suburban general practice in a relatively prosperous city. Patients were aged 65 or over, living at home, and were prescribed 10 or more regular items of medication. The sample reflects diversity of age (65-94); gender (11 men, 13 women); socioeconomic status; number of items of regular medication; co-morbidities. We excluded patients unable to speak adequate English. All patients gave written informed consent to participate in the study.

Each patient constituted a 'case'. We followed our participants' lives and experiences over an 18– 24 month period of ethnographic observation involving a range of data collection activities:

- 24 in-depth narrative interviews using Wengraf's biographic-narrative-interpretive method [20], eliciting a narrative with one opening question ("*please tell me the story of your life since you were first advised to take medicines*") and using the participant's account to elicit further narratives.
- 23 in-depth interviews focused on participants' medicines and medicines practices
- accompanying participants to selected clinical consultations (n=29), 18 of which were video-recorded

For peer review only - http://bmjopen.bmj.com/site/about/guidelines.xhtml

- cultural probes (photographs, body maps, diaries and imagined ‘wishful thinking’ conversations) which encouraged participants to depict their lives with their medicines) (14 participants)
- regular ‘follow up’ conversations (258 telephone calls; 66 home visits)

All interviews took place in participants’ homes. Two researchers (NF, a social anthropologist and DS, an academic GP, both experienced ethnographers in healthcare settings) undertook data collection and analysis. Two patients withdrew from the study after 8 and 11 months of follow-up.

Analysis

Fieldnotes and interview transcripts were shared between the researchers and we maintained a shared digital reflexive journal using Evernote® to document analytic memos and ongoing analytic insights, in addition to regular data analysis meetings. We used QSR NVivo 12 qualitative data analysis software to manage the data,[21] and adopted an interpretive approach to analysis, drawing on practice theory,[22 23] with a focus on assemblages of people, technologies, artefacts and their interconnections in context. All names reported in this paper are pseudonyms, and case narratives have been adapted to assure anonymity of research participants.

Patient and Public Involvement

We have an online patient panel of five members and a project advisory group with 11 members: lay chair; academics; health professionals; representation from Age UK; two patient members. Patients were involved in: proposal development; designing participant materials/project website (www.polypharmacy.org.uk); application for ethical approval; project launch event; piloting interviews; study design and conduct.

For peer review only - http://bmjopen.bmj.com/site/about/guidelines.xhtml

STUDY FINDINGS

Medications are much more than material objects with physiological effects; they are also representations that carry meanings and shape social relations as they evolve in conjunctions with individuals and collectivities.[9]

We begin by presenting an ethnographic account of the professional work of organising polypharmacy. This account focusses on the preparation of multi-compartment compliance aids (MCCAs or 'dosettes'). These were dispensed to four of our 24 research participants at the outset of our study; six participants received them by the end of our ethnographic follow up. Typically dosettes are dispensed to patients who are having difficulty managing their medicines independently. We spent 140 hours engaged in fieldwork in community pharmacies, where the scale and complexity of this 'backstage' organisational work became apparent.[24] Table 1 presents a brief description based on one community pharmacy site (for a more detailed account of our observations in community pharmacy see *[reference to Fudge, Swinglehurst BMJ Open 2021, in press]*).

Table 1. Ethnographic account of dosette preparation in Willow Pharmacy

Willow Pharmacy is the 'dosette hub' for the Woodland Independent Pharmacy group. The work of preparing dosettes is called '*being on production'* and is a technology-supported routine involving a full-time dosette robot and its human co-workers, typically three pharmacy technicians, also working full-time to ensure supply keeps up with demand. The robot cannot work unsupervised and needs constant attention. Demand for dosettes is increasing every month, as displayed on a hand-written poster on the wall in the '*production area*' where a running tally is kept. The pharmacy technicians are engaged in a constant, well-coordinated round of stocktaking, '*de-blistering*' drugs, '*replenishing*' the robot and running each production session. De-blistering is the process by which the staff prepare the medicines for the robot, as it cannot process medicines from their original packs. Pills are '*popped*' out, one by one, from their blister packaging into containers which slide into the robot. Some staff use a de-blistering machine for this, pulling a large metal lever to press the pills out of their packets into interim containers for checking by the pharmacist prior to replenishment. Others do it manually. Technicians work at speed and use a wide range of technologies including: computer programmes; Excel spreadsheets, often printed out; written log books; printed cards; scribbled post-it notes. All contribute to maintaining the smooth flow of routines and a sense of predictability as they generate dosettes in 4-weekly batches ready to stack on the shelves awaiting final checking. Medicines are referred to as '*fast lines*' (i.e. there is a lot of demand for them by the pharmacy's clients) or '*slow lines*' (i.e. less demand). This categorisation is an important component of decision-making regarding whether a particular medicine warrants inclusion in the robot. '*Slow lines*' may be designated '*externals*' (meaning that the medicine is added to a dosette box by hand once the robot has done its job). The integrity of medicines may be at risk if they are left out of their packaging for too long. Expensive lines are also dealt with as '*externals*' so that the pharmacy can remain agile in its

For peer review only - http://bmjopen.bmj.com/site/about/guidelines.xhtml

response to market price fluctuations. Despite everyone's best efforts to rationalise production, there are inevitably plenty of exceptions and contingencies to deal with. We often see filled dosettes recovered again from the shelves as staff attend to unanticipated changes in patients' prescriptions. This entails a painstaking process of using tweezers as a tool to isolate, break off and remove the backing from relevant cells of the dosette, then carefully removing and/or adding the relevant medicines, sealing the cell up again and attending to all the associated paperwork and electronic documentation. If the technician in the '*dosette corner*' receives too many requests for changes of this kind to the contents of dosettes in quick succession, the job quickly becomes very stressful.

This account offers some insight into the scale and complexity of the organisational work that polypharmacy presents and the range of professional considerations that are brought to bear as routines are developed to ensure that work proceeds as swiftly and safely as possible. It contextualises polypharmacy as the locus of *work.* In the remainder of this paper we consider the organisational work of our patient participants. We focus on patients who were dispensed all of their medications in their original packaging, where the burden of organisational work sits squarely with the patient. At the outset of our study 20 of the 24 participants received all their medication in original packaging.

Many participants expressed a sense of obligation and resignation with regards to taking medicines 'as prescribed', often expressing medicine-taking as a non-negotiable aspect of their lives. At the same time, they readily adapted their regimens to make them manageable. Their organisational efforts reflect a balance of these two broad commitments.

> *I have to take them, that's is. I have to accept it…I mean if I was on less, yeah, I'd be happy, but I'm not, so I just have to take them and that's it*
>
> (Biographical interview, Marco, DS)

> *I'm supposed to take that [carbocisteine] two tablets three times a day. I don't. Morning and evening. I usually forget the afternoon one, and it's possibly insufficient, but I have to balance one inconvenience with another inconvenience.*
>
> And later he goes on:

For peer review only - http://bmjopen.bmj.com/site/about/guidelines.xhtml

It [taking medicines] always takes place after breakfast, although breakfast can be anything between eight and ten. I know that I should be taking them [the 'morning' tablets] at a specific time each day in preference, but in life it doesn't always happen like that.

(Medicines interview, Charles, DS)

Participants invested considerable time and effort into organising their medicines into their lives, motivated by their striving to adhere to their medicines regimens:

I'm actually very methodical about it and I almost never miss any, which I believe is quite unusual, but it's because I've got this routine that I feel comfortable…it's been going on exactly the same for a number of years.

(Medicines interview, Elaine, NF)

Everything's in the kitchen…when they're on top of the microwave, I can't forget them.

(Medicines interview, Maria, NF)

I try to be organised, because it's easier to do that than getting stuff out every day. I'd find that very confusing.

(Medicines interview, Marian, DS)

When I ask Charles if he will show me his medicines he gets up quickly from his chair and moves towards the kitchen. I don't feel able to follow him there. He reappears with a little tray with a lip round the edge about an inch high. In it there is a very neatly organised set of medicine boxes lined up, with some sachets of Laxido [a laxative] propped up along one side. It looks very orderly and I wondered how he managed to find a tray that is such a perfect fit for his medicines boxes, or indeed what would happen if he were to stop half of them as they wouldn't tessellate and hold their place quite as they do in this neat arrangement. There is one open strip of capsules adjacent to the Laxido sachet, but otherwise everything is kept in its original box. He tells me that

For peer review only - http://bmjopen.bmj.com/site/about/guidelines.xhtml

they are kept in a kitchen cupboard and later when I switch on my audio-recorder for an interview he expands: "When we have guests or family or something, I'm told to put the bloody things away and not to display them and show off, and that sort of thing". Charles grimaces as he explains that his wife, a retired GP, does not like his medicines to be visible. He goes on to say that his wife thinks they should be kept discretely out of view. As he talks me through his medicines he lifts up the little tray from the coffee table onto his lap and goes through them meticulously, one by one, though he is careful to tell me that he is approaching them in 'no particular order'.

(Fieldnotes, home visit to Charles, DS)

These quotes and ethnographic notes point not only to the ways in which space, time and physical arrangements are harnessed to routinise and support medicines-taking but also how efforts at organising are a considered social performance. Our participants varied in the extent to which their medical complaints were made visible through their medicines to others who might visit the home. This finding resonates with the work of Palen and Aalokke who studied medicines management amongst elders in assisted living apartments in Denmark.[25] A key point of difference is that in the Danish study the participants received at least daily visits from a mobile health care worker who collaborated with elders in their medicines management. In our study home visits by health professionals were rare, even for our six housebound patients. Patients devised their own strategies and routines, although this often involved considerable resourcefulness and collaboration with others.

Given the number of items (10 – 30) and variety (tablets, creams, eye drops, injectables, inhalers and inhaled oxygen) of medicines prescribed to participants in our study their medicines regimens were inevitably very complex and needed careful organising. For example, one study participant consumed 21 tablets every morning, 15 tablets every evening and various 'as required' medicines in between; seven of our participants were prescribed 15 or more different items of daily medication. Despite the complexity of this work it was difficult to persuade our participants that there may be value in us learning about it as researchers. Whilst it was clear to us that they had given much thought to how to integrate their medicines into their lives, they mostly regarded their medicines as uninteresting, routine, normal, mundane

For peer review only - http://bmjopen.bmj.com/site/about/guidelines.xhtml

day-to-day business, but nevertheless '*a nuisance'*. Unlike their medical histories, which they often recounted in detail and in a well-rehearsed manner, they often struggled to articulate their approaches to organising and consuming their medicines ('*I just take them!*'). In part this may reflect the extent to which their routines had become embodied and successfully integrated into daily life; they no longer required ongoing careful consideration.

We now present three 'telling cases',[26] vignettes distilled from more detailed narrative case studies of each study participant which illuminate patients' hidden work of organising medicines into their lives in their efforts to adhere to complex prescribed medication regimens. These cases are not selected on the grounds of typicality but for their capacity to situate polypharmacy within a social context and challenge normative biomedical conceptualisations of polypharmacy. They speak to new ways of knowing the phenomenon of polypharmacy as it shapes the lives of patients experiencing complex multimorbidities. See Table 2 (Marco); Table 3 (Jackie) and Table 4 (Zac)

For peer review only

For peer review only - http://bmjopen.bmj.com/site/about/guidelines.xhtml

Table 2. Vignette of Marco

Marco is a retired chef. His narrative begins over 10 years ago at age 60 when he was diagnosed with diabetes and then with a '*cardiac problem*' which led to him having major surgery. He lives in an owner-occupied flat on the top floor of a low rise block in a deprived inner city area. We usually sit outside on his covered balcony when I visit; he smokes cigarettes and sips '*real*' coffee ('*life's little luxuries'*) as he talks.
He tells me about his blackouts, painful joints, lack of youthful energy and strength, fingers which get sore from finger prick tests (which he tactfully avoids except in the week before his diabetes check-up), and his warfarin levels which '*go up and down like a bloody yo-yo*'. He has no upper teeth and his dentures don't fit as he is awaiting dental treatment, so despite his fondness for cooking, eating is difficult.
Marco displays a sense of resignation about his ill health ('*I don't bother the doctor with everything – what can they do?*'). He is prescribed 15 items of medication but has not seen his GP for three years, although he attends the surgery regularly for nurse-led diabetes checks and warfarin monitoring blood tests ('*a nuisance*'). His medicines mean '*survival…I have to take them for life to keep me alive'* but at the same time they '*get on my nerves'* and '*I wouldn't take any of them by choice…but I don't have any choice*.'
Marco's pharmacist offered to package his medicines into a MCCA and deliver them direct to his flat, but he declined:

'*Sometimes you're offered a service but it doesn't mean you really need that service, you know what I mean? I can do it myself, it gives me a little exercise…it can wait a little while before I need the service…I can do it myself now*.'

He continues:

'*There's a couple of people in this block I noticed they have it [their medicines] delivered; they have this delivered, that delivered, when they're capable and it would be good for them to go around there and get it, you know?'* He goes on: *'sometimes it's just laziness.'*

I soon learn that Marco's friend Vicky helps him organise his medicines, though he tells me repeatedly that he could easily manage himself. Vicky is in her 40's and visits every day; she sometimes answers the phone when I call. She prepares his Do-It-Yourself [DIY] cassette-type MCCA on Sundays at a frequency that depends on the results of Marco's warfarin monitoring [International Normalised Ratio or INR]. If his INR is stable she may prepare four weeks at once. After all, Marco says '*you don't want to be undoing it all again do you? It would be nuisance*'. Vicky also prompts him to order his medication from his GP using an online service when supplies are low. Marco says he knows '*roughly*' what his tablets are, although a video-recording of one of his visits to the GP casts some doubt on how well he knows them by either name or purpose. Warfarin and insulin are the only medications he ever refers to by name – the others he knows by condition (diabetes, blood pressure), shape and size (the '*big one'*), time of day (the '*night one*', or organ ('*heart*'). This knowledge is adequate for his organisational purposes.

Marco explains that the DIY MCCA he uses currently is bigger than previous models he has tried - his medicines outgrew the smaller versions. The cassette consists of a stack of seven rectangular boxes labelled by day of the week. He takes the 'Monday' box from the bottom, causing the others to slide downwards within the supporting rack. There are 12 pills distributed across the four cells but each day he shifts the 'midday' ones that Vicky has prepared into the 'morning' cell (any time from 10 am until 1pm, it turns out) and shifts the 'teatime' ones into the 'evening' cell; two rounds of medicine-taking is easier than four. It seems that Vicky feels obliged to organise them as the prescription dictates, leaving Marco free to take responsibility for his personal re-organisation. He tries to take his evening medicines 20 minutes before eating but if he forgets - which he admits he

sometimes does - he takes them two hours after eating '*or thereabouts*'. He finds it '*very, very annoying'* that his medicines change in shape and colour so often, especially as he is colour blind and colours are confusing at best of times. His insulin pen sits on the coffee table, with extra supplies in the fridge. He keeps three inhalers in a 'Man Tin'. There is a glyceryl trinitrate (GTN) spray by the bed '*just in case my heart starts messing around*' and he adds '*it comes out with me all the time*'. In addition he keeps a basket in the bathroom where:

'*I do accumulate a little surplus, but I try not to, not so much, you know, just a little bit so it gives me an extra three or four days, besides what they give me, you know?.. But I don't have a massive supply, because what happens is if she [the GP] changes one of them in strength and they won't take the tablets back or anything back… only a few, not stocking up, you know.*'

He retrieves his warfarin paperwork which is tucked behind an ornament on the mantelpiece. He points to the date of his next appointment in one weeks' time. He explains he has to take his '*yellow book'* to the surgery every time he needs a prescription for warfarin; this requirement doesn't align well with the electronic routines now in place for ordering medicines online and transferring prescriptions between his GP and pharmacist.

On a later visit, Marco shares a poignant story about Vicky. When he met her a few years ago she was unable to manage her money, was overspending and not caring for herself. Marco bought her a tin, with two compartments – one for '*this week's*' money and one for '*next week's*' money – a kind of 'financial compliance aid' and has helped her '*get on her feet'* financially. Marco told me that he had advised Vicky that '*it is easy to live a life in which you just don't care about anything. It is harder to live a life in which you care.'*

For peer review only - http://bmjopen.bmj.com/site/about/guidelines.xhtml

Table 3. Vignette of Jackie

Jackie was diagnosed with a lifelong neurological condition as a teenager when she spent a year in hospital unable to walk. Her complex biography includes a stillbirth, a prolonged episode of severe loss of vision from which she recovered, numerous operations, diabetes, domestic abuse, two marriages. Now in her 70's she has ongoing chronic pain.<br><br>Jackie lives in social housing with her son and is prescribed 11 items of regular medication. She begins our interview by dropping her medicines accidentally on the floor. It is not unusual for us to find pills on the carpets when we visit our research participants; they are often clearly beyond their reach. With some difficulty Jackie manages to retrieve them and explains '*I take a metformin and something that goes with the metformin beginning with an 'A' – it's on my list somewhere – they go together in the morning*'. She refers to the '*thing that goes with the metformin*' four times during our interview and I conclude this is how she 'knows' and remembers it, though I later learn it is also '*the orange thing*'. She tells me '*I like to get them down my throat as quick as I can, because some of them have got some horrible taste to them…powdery, at the back of your throat, they lay there*'. She explains that the bad taste stays for about an hour. She goes on: '*In the evening, I take a metformin after a meal, on its own, and then - about no later than 10 o'clock - I take a co-codamol and carbamazepine and something beginning with 'E' which is a cholesterol tablet. And then just before I go to bed I take my diazepam to stop me shaking in the night*'. Through the day she is also guided by her pain, adjusting her medications '*according to how the pain is*' and she has other medicines which she takes to reduce the '*acid*' – a symptom she attributes to '*taking so many of them* [medicines]'. And then there are the '*terrible'* eye drops which she cannot manage independently because of the effects of MS on her hands:<br><br>*'I can hardly get them into my eyes. It's terrible. My son has to do it, because whenever anyone comes near my eye I shut it. And I said 'how long's that for? and she [the GP] said 'for life.'*<br><br>She thinks she is supposed to have her eye drops four times a day but as her son goes out to work at 06:45 there is no one to help with them during the day. A neighbour has offered but Jackie doesn't want to '*keep bothering people*'.<br><br>Jackie has a faded, well-used, Do-It-Yourself MCCA - one compartment per day, the days of the week now barely visible on the lid. David, her son, fills it every Sunday. She explains that she has got it out for me to see, but usually it's kept '*out of the way*' in the kitchen. Each compartment contains eight tablets (five separate items of medication). Jackie selects her tablets from each single compartment four times per day, choosing them by shape and colour. She is unsure of some of their names but, like Marco, she recognises them by their material properties. She is more than capable of filling this box herself, but David takes pride in helping with this crucial technical aspect of Jackie's care and she is grateful that '*I've got somebody keeping an eye on me*'. His role in helping her with her medicines management affirms his responsibility towards her as her official carer, alongside the more personal, hands-on aspects of her care, such as turning her most nights when she calls for him as she gets stiffness and spasms in her legs.<br><br>Although it is fiddly, Jackie manages to empty her 'as required' medicines (she calls them '*her spare ones*') from their blister packaging herself, and puts them together in a separate small '*spare tin*': co-codamol for pain, prochlorperazine for dizziness and omeprazole for indigestion (a pill which degrades outside its original packing). Like her regular medication in her DIY MCCA the different '*spares*' are mixed together. When we visit, this tin is in the kitchen, but it accompanies her to bed at night and fits neatly in her handbag for trips outdoors.

For peer review only - http://bmjopen.bmj.com/site/about/guidelines.xhtml

In addition, Jackie has a basket under her bed where she keeps tablets in their original packs. This is where she places medicines when they are delivered to her house by the pharmacy, and she tries to anticipate her needs by staying about one month ahead. She monitors her basket daily when she gets up and when she goes to bed, and calls the pharmacist on her mobile phone direct from her bed when she recognises her supplies are low. By keeping them under her bed and checking them daily she knows she won't '*get to a stage, I'd think Oh My God, I've got no tablets*'. She tries to strike a compromise between running out of medication and falling foul of the rather stark warning on the repeat prescription list she has from her doctor's surgery which reads: '*Don't stock-pile medicine at home – only order what you need*'. Like her MCCA, she keeps these medicines '*out of my way all day because they are upstairs*'. She prefers her medicines to be out of sight when she is not dealing directly with them.

Jackie speaks fondly of her local pharmacy and says they '*know me, because they call me Jackie, so they know me really well*'. But she complains that '*they* [the medicines] *never all run out together. It's a nightmare. I feel as though I am permanently ringing up the chemist for my tablets*'. We ask Jackie if there is one thing she could change about her medicines what would this be. Without hesitation she replies '*Not take any of them!*'

For peer review only - http://bmjopen.bmj.com/site/about/guidelines.xhtml

Table 4. Vignette of Zac

Zac, who is almost 90 is a retired secondary school teacher. He has lots of friends, is active in his church and serves his local community by running errands and cooking for people frailer than he. He tells me he derives great satisfaction from helping others. He adopts a dutiful, independent approach to his medicines and often talks about being a ‘good’ patient – ‘*my doctor tells me that I'm one of her best patients because I seem to do the right things, eat the right things and take the medication’*. He tells me that people cannot believe he is the age he is: *‘It is my education and my faith that have got me through…and I look after myself*’.

He keeps a three-month supply of medicines in a carrier bag by his armchair in his living room. Every Saturday, without fail, he organises his medicines for the following week. He lines up his morning and evening tablets, and puts them into 14 separate small plastic bags, wrapped inside tissue paper. He then puts them into his trouser pockets: left pocket for morning tablets and right pocket for evening tablets. He keeps a whole week's supply here, taking a packet from each pocket every morning and evening. The system works well for Zac. His medicines are high priority, kept close to his person and his busy lifestyle need not interrupt his medicines-taking - nor vice versa; he can keep religiously to his schedule wherever he happens to be.

Like Marco, Zac has been offered an MCCA and has resisted (‘*as long as I have my faculties*’). He heard on the news that people make mistakes with the doses of medication in these boxes; he doesn't want anyone ‘*mixing things up’* and his ‘*brain is turning over well*’.

A few months later, Zac is admitted to hospital. Some of his diabetes pills are stopped, he is advised to start insulin injections and he now receives a weekly MCCA. No one discussed the MCCA with him and he is unhappy about it, protesting ‘*while I am capable I want to do it myself*’. He now feels ‘*on edge’* every Wednesday and stays in his flat waiting for his medicines to drop through the letterbox. On one occasion they did not arrive until after 7pm, after his pharmacy's advertised closing time and he became extremely worried.

On another, he noticed the ‘*cholesterol medicine*’ was missing. In our cultural probe activity he explains that he wishes he could have a conversation with his GP about going back to his old system, adding ‘*I want to be in control of my own destiny*’. He wants to be free to go out and dislikes the weekly round of anxiety that he may run out of pills. He walked to the surgery several times hoping to secure an appointment to discuss this, only to be told there were no appointments.

Several months passed, before we observed a consultation between Zac and his GP. Zac explained ‘*I'm not very happy with the dosette box… they restrict me in my work and my movement.*’ The GP explained that Zac could have more than one box delivered at a time and promptly arranged this with the practice administrator responsible for MCCAs. Zac continued cautiously: ‘*I'm not particularly keen on dosette boxes. I'm quite careful about medicines’* but the GP did not get to the bottom of Zac's concern.

In conversation after the appointment, Zac explained that pharmacists can make mistakes with the medicines ‘*so you still have to check that they've done it properly’*, suggesting that he did not feel relieved of the organisational burden but had lost the sense of control and freedom that he enjoyed before. He complained that sometimes he can't recognise the tablets now because the brands change and went on to say that people who like dosettes are ‘t*aking the easy route*’.

For peer review only

We realise his diligence is being undermined and he speculates '*maybe they thought now I'm over 80 I can't manage*?' He tells us he will try out the '*four weekly dosettes*' then go back to the GP again and ask '*why I even need to be on them [the dosettes]*'. Several months later in our final discussion with Zac, before the end of our data collection period, he had yet to have this conversation with his GP.

These three cases convey a 'thick description' [27] of the nature and complexity of our study participants' medicines practices. Drawing on these cases as exemplars from our wider dataset we now synthesise these accounts to identify some key areas of shared experience and sense-making amongst our participants.

Jackie's account shows her days beginning and ending with a concern to monitor her medicine supplies, and the vignettes describe how these three participants have devised weekly temporal patterns of organising their DIY-dosette boxes to support daily routines. In Zac's case, trouser pockets stand in for boxes (he referred to his pockets as 'boxes' throughout one of our interviews). Zac's system allows him to adhere closely to taking his medicines at the prescribed times, which is a high priority for him. Maintaining his integrity as an active, helpful, independent, good citizen trumps any concerns he has for the integrity of the medicines in the storage conditions provided by his pockets. For Marco and Jackie, the preparation of the DIY-dosette boxes by Vicky and David, respectively, are important displays of reciprocity and part of a shared biography. In the case of Marco and Vicky, their mutual acts of organising form a tangible and important part of their ongoing support of each other.

In all cases, patients are balancing the perceived 'non-negotiable' requirement to take medicines with their desire for control over *how* they organise them into their lives. What emerges is inevitably a compromise. It is very difficult, perhaps impossible, for patients who are prescribed ten or more separate items of medication every day to take them strictly as prescribed, even when (as in our study) they express a clear intent to do so. As previous scholars have shown, there are practices of resistance,[28] and self-regulation,[29] within an overall context of reluctant acceptance of medicines as a way of life. All of our participants regard their organisation of their medicines as an expression of their desire to retain independence as far as this is possible, and to 'manage' themselves in a context of

For peer review only - http://bmjopen.bmj.com/site/about/guidelines.xhtml

increasing dependency. Marco wants to avoid '*bothering*' his GP; Jackie does not want to '*bother*' her neighbour about her eye drops. They all have strategies to ensure they do not run out of medicines.

Both Zac and Marco resist professional efforts to take over organising their medicines through the use of pharmacy-prepared MCCAs. They support this resistance with claims to cognitive capability. Marco's narrative points to the possibility of future decline when he may need to make use of the MCCA '*service*' but for now he constructs his identity as a capable, responsible user of both GP and pharmacy services who is prudent in ensuring he accumulates '*only a little surplus*' to avoid needless waste within a resource limited health system. Zac's persistent efforts to retain this position are ultimately thwarted.

DISCUSSION

Our study illuminates the organisational work that patients with multimorbidity do as they strive to adhere to their medicines in situations of complex polypharmacy. Polypharmacy requires organising, whether it occurs in the community pharmacy or at home. In both places this is demanding, time-consuming, and emotionally laden work. When medicines are prescribed, work is prescribed, but the nature, scale and complexity of this work usually remains invisible and unknown to the prescriber, and is rarely discussed during formal processes of medication review with patients. This work includes: practical acts of organising; surveillance of self and supplies; creative workarounds and experimentation with prescribed regimens; evaluation and management of personal priorities; anticipation and preparedness; management of self-presentation; and collaboration with carers and other professionals.

Previous research has identified households as 'hybrid centres of medication practice' where medicines are assimilated and many different forms of knowledge and expertise are brought together.[30] The key focus of our study is on individual patients, rather than households, but our findings show the extent to which patients' practices are embedded in, and shaped by, social context and relational networks. To our knowledge, our focus on older patients prescribed ten or more items of medication is unique. Our longitudinal ethnographic study design has enabled us to locate polypharmacy within these participants' biographies and lived experiences.

For peer review only - http://bmjopen.bmj.com/site/about/guidelines.xhtml

The ‘ordinary’ act of organising medicines emerges as a highly complex, deeply embodied and socially negotiated phenomenon. This work is typically regarded by the patients who are engaged in it as unremarkable, mundane and taken-for-granted – a ‘*nuisance*’. The ethnographic gaze unpacks the scale and scope of this ‘nuisance’ and brings into sharp focus the connectedness of medicines to meanings, social identities, practices and relationships that extend far beyond the technical and biomedical concerns which are typical of the clinic.

Pharmacy-prepared MCCAs or dosette boxes are often offered as one way of addressing the challenges of complex polypharmacy; approximately 64 million are issued in England per year.[31] Professional bodies discourage the use of MCCAs.[32 33] Royal Pharmaceutical Society (RPS) guidance clearly states: ‘*The use of original packs of medicines with appropriate support is the preferred option of supplying medicines in the absence of a specific need for an MCCA as an adherence intervention’.* The range of concerns includes but is not limited to: dispensing drugs in a MCCA is an unlicensed use of medicines; the limited evidence available (mostly derived from care home settings) indicates a lack of patient benefit;[32] many medicines become unstable when removed from their original packaging (and drug manufacturers are not required to test the stability of medicines repackaged in a MCCA);[32] patients may lose confidence about their medicines; scope for waste when prescriptions change. Pharmacists have legal responsibility for assessing a patient’s need for a MCCA and there have been recent calls to regulate pharmacists more heavily, to improve standards of MCCA practice and reduce the number issued.[31]

In this paper we have focused primarily on patients who are dispensed their medicines in their original packaging, as recommended in professional guidance (albeit Zac, like three other participants in our study went on to receive a MCCA part-way through our follow-up). It highlights some of the parallels between the work of the pharmacy in preparation of MCCAs and the work that patients must undertake when they are responsible for medicines which are dispensed in large numbers in their original packs. All of our participants devised ways of organising medicines that they could accommodate within their lives, often using DIY-MCCAs and often exposing their medicines to some of the same risks that underpin professional concerns about MCCAs (e.g. the risk of medicines losing their integrity when removed from their original packaging).

For peer review only - http://bmjopen.bmj.com/site/about/guidelines.xhtml

Calls to improve standards of MCCA practice are welcome.[31] However the goal of reducing their use in favour of dispensing medicines in their original packaging may fall short of the mark by failing to address the most complex issues at stake. The burden and risks of 'organising' polypharmacy in the service of medicines adherence always fall somewhere; the work does not go away. Polypharmacy in the context of complex multimorbidity is rarely, if ever, 'evidence-based', even when a robust argument can be made for the prescription of each individual item.[34] Combinations of drugs prescribed according to 'single disease' guidance can be both disruptive and dangerous in this context.[5 35] Unless the professional gaze shifts intentionally towards finding ways of tackling polypharmacy itself, the 'hidden' burden of organising polypharmacy will always fall somewhere, and will always carry risk.

For peer review only

Funding statement

This article presents independent research funded by the National Institute for Health Research (NIHR) through a Clinician Scientist Award (DS). In addition NF was (in part) supported by the National Institute for Health Research (NIHR) Collaboration for Leadership in Applied Health Research and Care (CLAHRC) North Thames at Bart's Health NHS Trust. The views expressed are those of the author(s) and not necessarily those of the NHS, the NIHR or the Department of Health and Social Care.

Competing interests

None declared

Author contributions

DS designed the study and secured funding. DS and NF jointly gathered and analysed data. DS wrote the first draft of the paper. Both authors revised and finalised the manuscript.

Acknowledgements

We would like to thank the staff and patients who participated in this study. We would also like to thank members of our Expert Advisory Group and our patient panel for their valuable advice, and colleagues at QMUL who commented on an early draft of the manuscript.

Ethics Approval

The project has ethics approval from Leeds West Research Ethics Committee (IRAS project ID: 205517; REC reference 16/YH/0462).

For peer review only - http://bmjopen.bmj.com/site/about/guidelines.xhtml

For peer review only

Data sharing statement

Our ethics approval was granted based on the anonymity of the individuals consenting to participate and our participant information sheets and consent forms reflected this. Our research participants have not consented to making our raw data publicly available

REFERENCES

1. World Health Organization. Medication Without Harm - Global Patient Safety Challenge on Medication Safety. Geneva: World Health Organization, 2017.
2. Guthrie B, McCowan C, Davey P, et al. High risk prescribing in primary care patients particularly vulnerable to adverse drug events: cross sectional population database analysis in Scottish general practice. *BMJ* 2011;342:d3514.
3. Payne RA, Avery AJ. Polypharmacy: one of the greatest prescribing challenges in general practice. *British Journal of General Practice* 2011;61(583):83-84.
4. Duerden M, Avery T, Payne R. Polypharmacy and medicines optimisation: Making it safe and sound. London: The King's Fund 2013.
5. May C, Montori VM, Mair FS. We need minimally disruptive medicine. *BMJ* 2009;339:b2803.
6. McCoy L. Time, self and the medication day: a closer look at the everyday work of 'adherence'. *Sociology of health & illness* 2009;31(1):128-46.
7. National Institute for Health Care Excellence. Medicines optimisation: the safe and effective use of medicines to enable the best possible outcomes. Available from: https://www.nice.org.uk/guidance/ng52015.
8. Royal Pharmaceutical Society. Medicines Optimisation: Helping patients to make the most of medicines. Available at: http://www.rpharms.com/promoting-pharmacy-pdfs/helping-patients-make-the-most-of-their-medicines.pdf2013.
9. Cohen D, McCubbin M, Collin J, et al. Medications as social phenomena. *Health:* 2001;5(4):441-69.
10. NHS Improvement. Network contract directed enhanced service. Contract specification. England: NHS, 2020.

For peer review only - http://bmjopen.bmj.com/site/about/guidelines.xhtml

11. Conrad P. The meaning of medications: another look at compliance. *Social science & medicine* 1985;20(1):29-37.

12. Cohen MD, Bacdayan P. Organizational Routines Are Stored As Procedural Memory: Evidence from a Laboratory Study. *Organization Science* 1994;5(4):554-68.

13. Whyte SR, Van der Geest S, Hardon A. Social lives of medicines. Cambridge: Cambridge University Press 2002.

14. Webster A, Douglas C, Lewis G. Making sense of medicines:'lay pharmacology'and narratives of safety and efficacy. *Science as Culture* 2009;18(2):233-47.

15. Dew K, Norris P, Gabe J, et al. Moral discourses and pharmaceuticalised governance in households. *Social Science & Medicine* 2015;131:272-79.

16. Suchman L. Plans and situated actions: The problem of human-machine communication. Cambridge: Cambridge University Press 1987.

17. Kim J, Whitney A, Hayter S, et al. Development and initial testing of a computer-based patient decision aid to promote colorectal cancer screening for primary care practice. *BMC Medical Informatics and Decision Making* 2005;5(1):36.

18. Huyard C, Haak H, Derijks L, et al. When patients' invisible work becomes visible: non-adherence and the routine task of pill-taking. *Sociology of health & illness* 2019;41(1):5-19.

19. Swinglehurst D, Fudge N. Addressing the polypharmacy challenge in older people with multimorbidity (APOLLO-MM): study protocol for an in-depth ethnographic case study in primary care. *BMJ open* 2019;9(8):e031601.

20. Wengraf T. Qualitative research interviewing : biographical narrative and semi-structured methods. London, Thousand Oaks, New Delhi: Sage Publications 2001.

21. QSR International. NVivo Qualitative Data Analysis Software [program] Version 12 2018 [Available from: https://www.qsrinternational.com/nvivo-qualitative-data-analysis-software/home.

22. Schatzki TR. Introduction: practice theory. In: Schatzki TR, Knorr Cetina K, von Savigny E, eds. The Practice Turn in Contemporary Theory. Abingdon, New York: Routledge 2001:1-14.

23. Nicolini D. Practice Theory, Work, & Organization: An introduction. Oxford: Oxford University Press 2012.

For peer review only

For peer review only - http://bmjopen.bmj.com/site/about/guidelines.xhtml

24. Goffman E. Regions and Region Behaviour. The Presentation of Self in Everyday Life. London: Penguin Books 1959:109-40.

25. Palen L, Aaløkke S. Of pill boxes and piano benches: "home-made" methods for managing medication. *Proceedings of the 2006 20th anniversary conference on Computer supported cooperative work* 2006:79-88.

26. Mitchell JC. Producing data: Case studies. In: Ellen RF, ed. Ethnographic Research: A Guide to General Conduct. London: Academic Press 1984:237-41.

27. Geertz C. The Interpretation of Cultures. New York: Basic Books 1973.

28. Pound P, Britten N, Morgan M, et al. Resisting medicines: a synthesis of qualitative studies of medicine taking. *Social Science and Medicine* 2005;61:133-55.

29. Pollock K, Grime J. Strategies for reducing the prescribing of proton pump inhibitors (PPIs): patient self-regulation of treatment may be an under-exploited resource. *Social science & medicine* 2000;51(12):1827-39.

30. Dew K, Chamberlain K, Hodgetts D, et al. Home as hybrid centre of medication practice. *Sociology of Health and Illness* 2014;36:28-43.

31. Yeung A. North East and North Cumbria Medicines Adherence Support Project, 2019.

32. Royal Pharmaceutical Society. Improving patient outcomes–the better use of multi-compartment compliance aids: Royal Pharmaceutical Society Great Britain, 2013.

33. London Medicines Information Service. Summary of Guidance and Evidence for use of Multi-Compartment Compliance Aids, 2019.

34. Armstrong N, Swinglehurst D. Understanding medical overuse: the case of problematic polypharmacy and the potential of ethnography. *Family practice* 2018

35. Swinglehurst D, Hjörleifsson S. The everyday ethics of burdensome polypharmacy. *Public Policy & Aging Report* 2018;28(4):113-15.

For peer review only - http://bmjopen.bmj.com/site/about/guidelines.xhtml

Open access Protocol

BMJ Open

Addressing the polypharmacy challenge in older people with multimorbidity (APOLLO-MM): study protocol for an in-depth ethnographic case study in primary care

Deborah Swinglehurst, Nina Fudge

To cite: Swinglehurst D, Fudge N. Addressing the polypharmacy challenge in older people with multimorbidity (APOLLO-MM): study protocol for an in-depth ethnographic case study in primary care. *BMJ Open* 2019;**9**:e031601. doi:10.1136/bmjopen-2019-031601

► Prepublication history for this paper is available online. To view these files, please visit the journal online (http://dx.doi.org/10.1136/bmjopen-2019-031601).

Received 10 May 2019
Revised 15 July 2019
Accepted 25 July 2019

Check for updates

© Author(s) (or their employer(s)) 2019. Re-use permitted under CC BY. Published by BMJ.

Centre for Primary Care and Public Health, Blizard Institute, Barts and The London School of Medicine and Dentistry, Queen Mary University of London, London, UK

Correspondence to
Professor Deborah Swinglehurst;
d.swinglehurst@qmul.ac.uk

ABSTRACT

Introduction Polypharmacy is on the rise. It is burdensome for patients and is a common source of error and adverse drug reactions, especially among older adults. Health policy advises clinicians to practice *medicines optimisation*—a person-centred approach to safe, effective medicines use. There has been little research exploring older patients' perspectives and priorities around medicines-taking or their actual practices of fitting medicines into their daily lives and how these are shaped by the wider context of healthcare.

Methods and analysis We will conduct an in-depth multisite ethnographic case study. The study is based in seven clinical sites (three general practices and four community pharmacies) and includes longitudinal ethnographic follow-up of older adults, organisational ethnography and participatory methods. Main data sources include field notes of observations in the home and clinical settings; interviews with patients and professionals; cultural probe activities; video recordings of clinical consultations and interprofessional talk; documents. Our analysis will illuminate the everyday practices of polypharmacy from a range of lay and professional perspectives; the institutional contexts within which these practices play out and the sense-making work that sustains—or challenges—these practices. Our research will adopt a 'practice theory' lens, drawing on the sociology of organisational routines and other relevant social theory guided by ongoing iterative data analysis.

Ethics approval The study has HRA approval and received a favourable ethical opinion from the Leeds West Research Ethics Committee (IRAS project ID: 205517; REC reference 16/YH/0462).

Dissemination Aside from academic outputs, our findings will inform the development of recommendations for practice and policy including an interactive e-learning resource. We will also work with service users to co-design patient/public engagement resources.

Strengths and limitations of this study

- ► Gathers rich qualitative data across community settings (the home; general practice; pharmacy) with a focus on how practices unfold on the ground and how different actors make sense of polypharmacy.
- ► Brings together analysis of patients' lived experience with analysis of organisational routines and professional practices.
- ► Uses innovative qualitative methodologies which generate more sophisticated insights than is possible with conventional approaches.
- ► Prioritises depth of analysis over breadth, increasing richness of understanding but limiting generalisability of findings.

INTRODUCTION

Polypharmacy is increasing. One Scottish study showed that about 20% of adults are dispensed five or more drugs (this doubled between 1995 and 2010) and about 6% of adults are dispensed 10 or more drugs (this tripled over the same time period).[1] There is growing concern that polypharmacy is one example of 'medical overuse', causing unnecessary burden, iatrogenic harm to patients and costly waste to health systems. Up to half of medicines prescribed for long-term conditions are unused.[2] This is often conceptualised as a problem of poor adherence[3–5] or as a failure of shared decision-making in the consultation.[6 7] The problem is assumed to be located within individuals' behaviours, with relatively little attention paid to the relationship between individuals and the wider social, organisational and institutional context within which their medicines practices play out.

Polypharmacy is often attributed to the ageing population; 65% of people aged 65–84, and over 80% of people aged 85+ experience multimorbidity.[8] But ageing per se is only one of many potentially relevant factors. For example, evidence-based guidance which informs prescribing is typically organised around a 'single-disease' model

For peer review only - http://bmjopen.bmj.com/site/about/guidelines.xhtml

BMJ Open: first published as 10.1136/bmjopen-2019-031601 on 22 August 2019. Downloaded from http://bmjopen.bmj.com/ on August 23, 2019 by guest. Protected by copyright.

and may compound rather than alleviate polypharmacy. One critic has described evidence-based medicine as a perverse 'monument to bias', built on its systematic exclusion of complex patients with multimorbidity from the trials that inform it.[9] Arguably, polypharmacy in the context of multimorbidity is rarely, if ever, evidence based, even when a clear 'evidence-based' argument can be made for individual items.[10] Achieving *medicines optimisation*—a person-centred approach to safe, effective medicines use[11–13]—is fraught with difficulty when the institutional context privileges the single-disease model and is increasingly concerned with the application of abstract, generalisable rules. This is further complicated by the increasing emphasis on what has been called 'risk factorology' and the quest to eliminate risk of disease altogether.[14] The societal context is increasingly one in which 'every responsible and rational citizen is expected to actively seek out and eliminate all possible risks to their future health and to consume medical technologies in order to achieve this aim'.[15] Pay-for-performance initiatives which embrace (and arguably constitute) aspirations to eliminate risk and uncertainty may also drive polypharmacy.

A recent study which examined temporal trends between 2000 and 2015 in the use of tests and investigations in UK primary care found that age-adjusted and sex-adjusted use of tests and investigations increased by a staggering 8.5% *annually*.[16] Although prevalence studies do not offer insight into the drivers of change, it seems reasonable to conclude that the parallel and dramatic rise in investigations and polypharmacy over a similar time period may be related phenomena. Privileging priorities for reducing risk of disease may contribute paradoxically to new drug-related risks—risks which increase with increasing age and with increasing numbers of medications.[17] 'Problematic' polypharmacy[13] arises insidiously, despite (and sometimes even because of) clinicians and patients striving to achieve best practice and acting with the very best of intentions. The elusive boundary between 'just enough medicine' and 'too much medicine' is unstable, contingent and socially constructed. It involves clinical judgement in the context of inadequate evidence and considerable uncertainty. Sinnott has referred to the work of negotiating this boundary as a process of 'satisficing'.[18] Doctors report settling for management that is satisfactory and sufficient given the particular circumstances of a patient—a combination of relaxing nationally recommended targets, compromise, hunch, guesswork and maintaining the status quo when patients appear stable.[18]

Polypharmacy presents a complex challenge for clinicians and health systems. It arises at the interface of patients, clinicians, diseases (and risk thereof) and involves a tangle of biological, cultural, technological, economic and sociopolitical dimensions. It is a 'wicked problem'.[19] One feature of wicked problems is their resistance to adequate definition. Polypharmacy tends to be defined numerically, with one recent systematic review identifying 138 different definitions.[20] But simple numerical definitions conceal much of the complexity that the polypharmacy concept evokes.[17] Rittel states that 'the formulation of a wicked problem *is* the problem'; wicked problems are ill-defined, not amenable to a quick-fix 'solutions' but at best '*resolved, or rather re-solved, over and over again*'. Wicked problems coalesce around important moral issues whereby there are acknowledged discrepancies between the state of affairs as it *is* and the state as it *ought* to be.[19] Ethnographic approaches are especially good at investigating complex issues such as polypharmacy and shedding light on ill-defined but troubling phenomena.[21] In this study, we will adopt an ethnographic approach and apply a range of innovative methods which focus on meanings, experiences and practices, as we encounter them in the lives of patients and professionals. This approach embraces polypharmacy as a wicked problem, offering 'new ways of looking' and thereby opening up potential new avenues for addressing polypharmacy in practice and policy.

AIM

The aim of the APOLLO-MM project is to improve patient care by producing 'practice-based' evidence[22] to inform medicines optimisation. We will do this by:

- Examining patients' experiences of polypharmacy to discern elements of high-quality 'medicines work' and those elements that might lead to harmful, wasteful or unnecessary polypharmacy.
- Improving understanding of how polypharmacy is sustained and/or challenged within and between lay and professional networks.
- Analysing consultations that include talk about medicines (including but not limited to formal 'medication review') to understand how medicine optimisation is negotiated in practice.
- Using participatory methods to elicit professional dialogue around polypharmacy and offer opportunities to discern the elements of good practice in medicines optimisation.
- Working with patients and professionals in a process of co-design to develop e-learning materials, recommendations for practice and public/patient engagement resources.

RESEARCH QUESTIONS

1. What is the patient experience of polypharmacy in multimorbidity?
2. What does polypharmacy mean for patients and carers?
   a. How are patients' lives with multimorbidity shaped by practices of managing medicines?
   b. How is the managing of medicines shaped by contingencies of living with multimorbidity?
3. How do the practices of patients/carers/health professionals and wider systems of care support 'appropriate' polypharmacy or challenge 'inappropriate' polyphar-

For peer review only - http://bmjopen.bmj.com/site/about/guidelines.xhtml

BMJ Open: first published as 10.1136/bmjopen-2019-031601 on 22 August 2019. Downloaded from http://bmjopen.bmj.com/ on August 23, 2019 by guest. Protected by copyright.

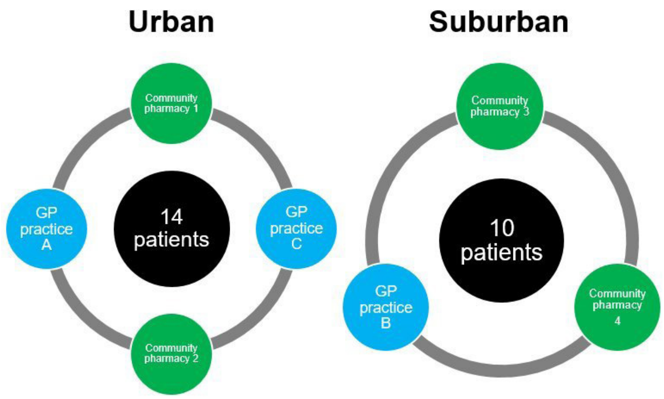

Figure 1 Study sites and research clusters. GP, general practitoner.

macy and with what consequences for the quality and safety of care?

4. How can insights from longitudinal patient case studies inform improvements in professional practices, service delivery and public awareness of polypharmacy?

METHODS

Setting and context

The study is a multicentre ethnographic case study involving seven clinical sites across two research clusters in England (one urban and one suburban cluster). The urban cluster comprises two National Health Service (NHS) general practitioner (GP) practices (Site A and Site C) and two independent community pharmacies located in an area of high socioeconomic deprivation within a large city, serving a population of diverse ethnicity comprising approximately 40% Asian patients. The suburban cluster comprises one NHS GP practice (Site B) and two independent community pharmacies, located in a small, relatively prosperous city serving a population which is 85% white Caucasian. The research clusters represent contrasting demographics; the urban cluster serves patient populations within the lowest (Site C) and second lowest (Site A) decile of the English Index of multiple deprivation score,[23] and the suburban cluster serves a population within the least deprived decile.

The GP practices were selected following expressions of interest from over 40 GP practices within the North Thames Clinical Research Network area, suggesting that the project resonates with the concerns of GPs on the ground.

We have recruited 24 patients, 14 in the urban cluster (seven each from Sites A and C) and 10 from our suburban cluster. The work of patient follow-up and organisational ethnography has begun. The GP practices and community pharmacies in the study share responsibility for prescribing and dispensing medicines to the study patients, allowing for multiple perspectives of polypharmacy. To date, we have recruited 40 professional participants for interviews, filming of consultations and participation in research workshops (see the Study design, methodology and approach to analysis section). The relationship between the patients, GP practices and community pharmacies is shown in figure 1.

Selection of organisational cases

Drawing on Stakes approach to organisational case study our selection of GP sites primarily reflects (1) *opportunity for learning*, rather than concerns about representativeness[24] and (2) diversity of geographical setting and population demographics. Our concern is to conduct an in-depth study of *particular* practices within these sites, adopting an interpretive approach informed by relevant social theory. We will create generalisable theoretical and conceptual abstractions (novel concepts to 'think with') rather than generalisable statistical abstractions. The ethnographic approach relies on the interest shown in the study by the potential participants, the willingness of staff at all levels of the organisation to allow meaningful access to the research team (eg, shadowing, observations, formal and informal interviews; access to relevant documents) and enthusiasm for the participatory element of the study within the context of already heavy workloads (see the Study design, methodology and approach to analysis section). In addition, we considered a range of pragmatic concerns relating to feasibility, location and likelihood of building sustainable working relationships for the duration of the study. The table 1 shows some of the characteristics of our recruited practices.

Sampling of patient participants

We have recruited patients aged 65 or over and prescribed 10 or more regular (repeat) items of medication, this being regarded a pragmatic marker of 'high-risk polypharmacy'.[13] Where patients were prescribed an item in more than one dose (eg, warfarin), this was counted as a single item. Medical devices and hosiery were excluded, but medicines in all forms (eg, oral, inhalers, injectables, topical creams, etc) were included. We adopted a purposive sampling approach, aiming for a maximum diversity sample across a range of attributes: age (ranging from 65 to 94); gender (11 men; 13 women); living circumstances (16 live alone of whom five in sheltered housing; eight with partners/family; four participants are housebound); socioeconomic status (home ownership and previous occupation as proxy indicators) and number of medication items.[10–17] All patients have two or more comorbidities. Twenty-one out of 24 patients are white Caucasian. We have excluded patients unable to speak adequate English, which means that sampling in our urban cluster is not representative of the local population. We are addressing this limitation in another study.[25]

Recruitment took place in general practice. GPs and nurses introduced the study to potential participants, issued participant information leaflets and sought verbal consent from patients to be contacted by a researcher (n=63). Thirty-six patients agreed to a home visit to discuss the study in more detail and 24 consented to take part. Patients lacking capacity to consent were eligible for inclusion on the identification of a personal consultee.

For peer review only

BMJ Open: first published as 10.1136/bmjopen-2019-031601 on 22 August 2019. Downloaded from http://bmjopen.bmj.com/ on August 23, 2019 by guest. Protected by copyright.

For peer review only - http://bmjopen.bmj.com/site/about/guidelines.xhtml

Table 1 Characteristics of recruited GP practices

	Site A	Site B	Site C
Cluster	Urban	Suburban	Urban
Patient population	c 11 000	c 13 000	c 14 000
GPs	5 partners; 5 salaried	4 partners; 6 salaried	8 partners; 3 salaried
Onsite clinical pharmacist at recruitment	No	No	No
Onsite clinical pharmacist for part of project duration	Yes	Yes	Yes
GP training practice	Yes	Yes	Yes
Deprivation*	Second most deprived decile	Least deprived decile	Most deprived decile
Ethnic diversity (estimated proportion non-white ethnic groups)*	Over 50% (over 40% of practice population are Asian)	14%	Over 60% (over 40% of practice population are Asian)

*Details from National General Practice Profiles produced by Public Health England Data Science.
GP, general practitioner.

A consultee is someone who, by virtue of their existing relationship with the potential participant, can advise the researcher about their participation in the project. One patient (with dysphasia following a stroke) has a personal consultee, their next of kin, who is their primary caregiver. We were unable to recruit any patients with dementia; we are addressing this using a different recruitment strategy in a parallel study which focusses specifically on patients with dementia[25]

Study design, methodology and approach to analysis

The figure 2 shows the methods and data sources for the study, which are designed to elicit and explore patient experiences and practices; professional experiences and practices and professional dilemmas. These are described in detail in the following sections.

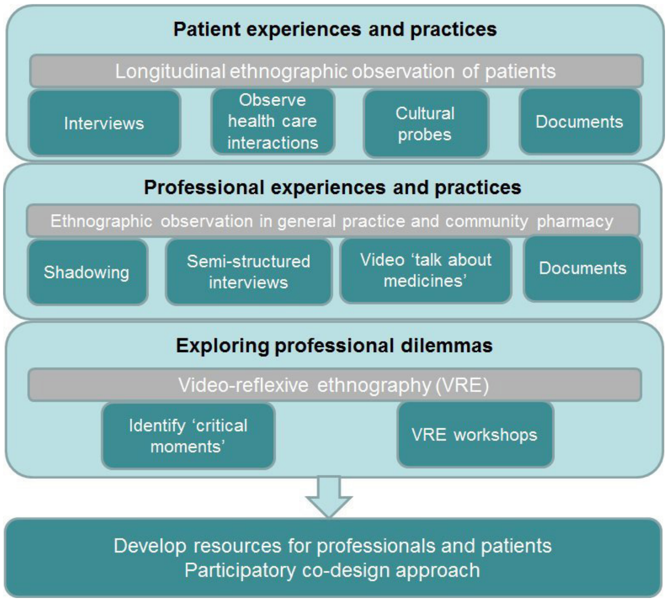

Figure 2 Methods and data sources.

Patient experiences and practices

We will follow patient participants ethnographically over 18–24 months, observing them at home and accompanying them to selected healthcare interactions in which they anticipate talk about their medicines (eg, at the GP surgery, pharmacy or hospital). We are conducting interviews with patients to elicit the story of their lives since they were first advised to take medicines, adapting Wengraf's Biographic-Narrative Interpretive Method.[26] This approach utilises a single question to induce narrative and invites participants to speak uninterrupted until their story is complete. After a short break (10–15 min), the researcher presents a series of further questions based on 'cue phrases' (the patient's own words) in the order the patient introduced them into their opening narrative, to encourage further in-depth narratives (particular incident narratives). On a separate occasion, we return to the patient's home to conduct an in-depth interview guided by a topic guide with a more explicit focus on medicines practices. We contact participants by telephone or home visit at approximately 4–8 weekly intervals to learn about their ongoing experiences and to identify occasions when we may accompany them to a healthcare interaction or video record a consultation. Every time we meet our participants, we take detailed ethnographic field notes, add to our reflexive journal and gather relevant documents (eg, discharge summary or lists of medication). This data enables us to build detailed patient case studies as we follow their lived experience and engage them in informal 'event-based' interviews exploring their care experiences.

A subset of patients are completing cultural probe activities as a way of depicting their lives. Cultural probes originated in the world of design as a way of tapping into peoples' creativity and offering research participants opportunity to engage in a research-related activity that is subjective, deliberately ambiguous, open

For peer review only - http://bmjopen.bmj.com/site/about/guidelines.xhtml

BMJ Open: first published as 10.1136/bmjopen-2019-031601 on 22 August 2019. Downloaded from http://bmjopen.bmj.com/ on August 23, 2019 by guest. Protected by copyright.

to interpretation, invites imagination and stimulates conversation.[27 28] The probes consist of a set of activities and a range of materials (eg, camera, pens, cards, booklets) with simple instructions which offer opportunity for improvisation. They encourage participants to think about their medicines and bodies in new ways and reimagine possibilities for how they might interact with health professionals. The creative outputs are used to stimulate an informal discussion between researcher and participant. Participants may invite a family member or researcher to assist if they wish. Cultural probes have been used successfully to incorporate older people's ideas into the design of social communities[27], and to illuminate the daily living needs of older people to inform co-production of assistive living technologies,[29 30] although it is recognised that some people may be unable to engage with them for a range of physical, emotional and social reasons.[29]

Professional experiences and practices

We will conduct ethnographic observations in three GP and four community pharmacy practices (see figure 1). Our interest is in understanding how health professionals conduct and make sense of their day-to-day work of prescribing, dispensing, negotiating, organising medicines for patients affected by polypharmacy. We will observe key routines such as preparation and organisation of multicompartment compliance aids (MCAs or 'dosette boxes'); repeat prescribing and repeat dispensing.

We will closely observe individuals at work (shadowing and asking participants to describe what they are doing as they do it to encourage the articulation of tacit knowledge); organisational routines and how they are constituted; the wider institutional context and documents relevant to the work we observe (eg, prescriptions, guidelines). We will also interview key professional participants across our sites with a focus on accounts of their working practices. We will hone in on consultations between clinicians and our study patients to examine healthcare interactions in detail by video recording consultations in which our participants anticipate 'talk about medicines'. The polypharmacy context is a very particular context for these interactions and we are interested to learn how polypharmacy is sustained, challenged, talked about and 'brought about' in these interactions.

Organisational routines are conceptualised as 'repetitive recognisable patterns of interdependent actions by multiple actors'[31 32] and can be regarded as sites of stability and as sites of potential for innovation and change.[33] When participants engage in routines they enact tacit knowledge and are involved in complex patterns of collaboration—involving people, artefacts, technologies—to get work done. Routines can be embodied, embraced, resisted and enacted with varying degrees of creativity and improvisation and varying attention to the particularities of local context.[34] Pentland and Feldman have conceptualised routines as being composed of:

- Ostensive routines: the generalised 'rules' or organisational scripts that guide the routine, of which there may be many instantiations.
- Performative routines: what people *actually do in practice* as they engage in the routine. Each instantiation of the routine is a unique performance.

Ostensive routines may be accessed through peoples' accounts of the work they do, while observation of repeated rounds of a routine gives insight into the many versions of the performative routine and the relationship between the performative and the ostensive aspects of the routines. When people engage in routines, they select from a repertoire of possible actions (enacting possibilities around which there are explicit or implicit constraints). Different people bring different knowledge, different expertise, different assumptions and different personal and organisational narratives to the enactment of the routine. An understanding of the patterns of possibility and constraints offers insights into wider organisation principles and values, and to an appreciation of complex organisational practices, such as how power circulates and how learning is done. The dynamic interplay between the ostensive and performative routines and the artefacts which are drawn on to support these routines can transform what has traditionally been regarded as mundane and ordinary organisational life (eg, the processing of a repeat prescription) into opportunity for 'thick description'[35] of the nature of organisational life and the wider context within which work is being done.[32 36] Continuities and discontinuities in the relationship between ostensive and performative routines may become manifest as puzzles, conflicts, moments of perplexity or tensions.

Recent literature on organisational routines has pointed to the need to pay greater attention to the relational nature of 'self' and agency in the performance of routines and in the dynamic processes that address *how routines become collective accomplishments* with recognisable shared collective characteristics (ie, how the 'ostensive' aspect of routines is constituted or brought about).[37 38] One approach which offers promise is to locate the study of routines within emerging 'communication as constitutive of organisation (CCO)' scholarship,[39–42] where communication is itself framed as performance (through conversation and text dialectic). Routines are then framed as communicatively constituted performances, there is greater emphasis placed on performativity, embodiment, dialogic inter-relationships and interest in 'strings of associations' that link actors (human and non-human) as routines unfold.[43] The nuance of communication gains ground—the emergence of organisational storylines, for example.[44–46] This approach aligns with our interest in patient narratives and our orientation to 'talk about medicines' as co-constructed accomplishment within the consultation.

Video reflexive ethnography (VRE)

VRE is an innovative approach to quality and safety research in healthcare[47–49] which encourages groups

For peer review only - http://bmjopen.bmj.com/site/about/guidelines.xhtml

BMJ Open: first published as 10.1136/bmjopen-2019-031601 on 22 August 2019. Downloaded from http://bmjopen.bmj.com/ on August 23, 2019 by guest. Protected by copyright.

of professionals to discuss and reflect on their working practice. In this study, selected video recorded footage of 'medicines talk' in our study patients' consultations will be shown to small groups of professionals across our research sites. VRE offers an opportunity for participants to negotiate shared meanings in their practice, engage with the complexity of their everyday work, and make visible—in new ways—aspects of their practice which have become habituated, taken for granted and 'invisible'.[49] It is a participatory approach in which researchers and practitioners work together to illuminate this complexity and foster dialogue that might not otherwise occur.[50] The footage acts as a catalyst, making implicit knowledge explicit through a process of reflection-on-action which renders the work (in this case, the practical work of 'medicines optimisation') understandable in new ways and open to new ways of re-imagining practice.[51] Importantly, the discussion may go beyond what is visible in the footage, the footage acting as a prompt to inter-professional exchange and peer-led discussion of professional concerns, dilemmas and opportunities. There is opportunity for 'exnovation' or innovation from *within* practice[48] and the cultivation of meta-discourses of practice[50] which can reframe everyday practices (eg, the 'medication review') by drawing out the systemic implications of what specific people know, do and say. By video recording the discussion, this meta-discourse can be teased out and worked with across VRE sessions, in ways which enable practitioners to iteratively reframe their work and negotiate new possibilities for action. Importantly, the collaborative process of co-constructing this meta-discourse in the VRE context may itself be a rehearsal space for new ways of relating and collaborating in the very doing of the work that is under scrutiny.

Participatory co-design

The findings from the research will feed into a process of participatory co-design, working in collaboration with both patients and professionals to design e-learning materials for professional education and patient engagement resources for patients. In this work, we will adapt processes of experience-based co-design[52 53] and 'future groups',[54] the latter being a reinterpretation of focus group methods which uses ideation tools and encourages speculation to imagine alternative approaches to care. We will collaborate with a design researcher to develop our research materials and findings to create bespoke ideation tools and props to facilitate imaginative participation and move towards exploring future alternatives to care prompted by the stories, data and findings from the earlier research.

Integrating analysis across diverse data sets

The project is situated within a broad 'practice theory' orientation and adopts a social constructionist orientation. Practice theory encourages an understanding of work as an accomplishment and fosters a curiosity about what is accomplished by whom and how. The organisation (eg, the GP practice or pharmacy) is understood as both the *site* and the *result* of work activities and the role of the interpretive researcher is to 'zoom in' on the details of particular practices and to 'zoom out' to discern the relationships and connections between people, routines, artefacts, spaces and technologies, for example.[55 56] As our analysis progresses, we will, in addition, draw on relevant social theory (eg, Burden of Treatment theory[57 58]) to explicate our findings.

This detailed ethnography will extend our previous research on repeat prescribing in general practice settings in four ways.[36] First, the institutional context has changed (one example being the implementation of the Electronic Prescribing Service, another being the incorporation of clinical pharmacists within some GP settings). Second, our research extends beyond general practice into community pharmacy settings and into peoples' homes and opens up a wider range of vantage points from which to observe the phenomenon. We are focussing our observations around the phenomenon of polypharmacy, recognising that polypharmacy emerges out of an arrangement of practices and is not a discrete entity that can be easily observed without appreciation of a wider social context. Finally, our research includes a commitment to 'working with' both health professionals and patients in a process of co-design to produce outputs which we hope will reflect what really matters to staff and patients on the ground.

PATIENT AND PUBLIC INVOLVEMENT

We have a project advisory group of 11 members including academics, health professionals, representation from Age UK, two patient members and a lay chair. Patients have been involved in the proposal development, design of participant materials and project website (www.polypharmacy.org.uk), our application for ethical approval, the project launch event, piloting of interviews, study design and conduct. We have an online patient panel of five members.

PROJECT MANAGEMENT AND GOVERNANCE

Our project is overseen by an expert advisory group (see above) and we report annually to the Heath Research Authority and our funder (National Institute of Health Research).

Site recruitment began in March 2017. Patient recruitment began January 2018 and was completed October 2018. Project funding ceases on 28/2/2021.

ETHICS AND DISSEMINATION

The project has ethics approval (IRAS project ID: 205517; REC reference 16/YH/0462).

Our findings will inform the following key outputs.

1. For clinicians: key recommendations for practice; interactive online e-learning resources.

For peer review only

BMJ Open: first published as 10.1136/bmjopen-2019-031601 on 22 August 2019. Downloaded from http://bmjopen.bmj.com/ on August 23, 2019 by guest. Protected by copyright.

For peer review only - http://bmjopen.bmj.com/site/about/guidelines.xhtml

2. For professional bodies: summary of findings to inform professional guidance.
3. For patients and carers: co-designed resources to inform patient and public understanding of polypharmacy, inform decision-making and improve consultations with health professionals.
4. For policymakers: a summary report of key findings.
5. Academics: research publications and conference presentations.

Acknowledgements We would like to thank the staff and patients who have agreed to participate in this study. We would also like to thank members of our Expert Advisory Group and our patient panel for their valuable advice.

Contributors DS designed the study and secured funding. DS and NF have both been involved in fieldwork and have both contributed to writing this paper.

Funding statement This article presents independent research funded by the National Institute for Health Research (NIHR) through a Clinician Scientist Award (DS). In addition NF was (in part) supported by the National Institute for Health Research (NIHR) Collaboration for Leadership in Applied Health Research and Care (CLAHRC) North Thames at Bart's Health NHS Trust. The views expressed are those of the author(s) and not necessarily those of the NHS, the NIHR or the Department of Health and Social Care.

Competing interests None declared.

Patient consent for publication Not required.

Provenance and peer review Not commissioned; externally peer reviewed.

Open access This is an open access article distributed in accordance with the Creative Commons Attribution 4.0 Unported (CC BY 4.0) license, which permits others to copy, redistribute, remix, transform and build upon this work for any purpose, provided the original work is properly cited, a link to the licence is given, and indication of whether changes were made. See: https://creativecommons.org/licenses/by/4.0/.

REFERENCES

1. Guthrie B, Makubate B, Hernandez-Santiago V, *et al*. The rising tide of polypharmacy and drug-drug interactions: population database analysis 1995-2010. *BMC Med* 2015;13:74.
2. Sabate E. *Adherence to long-term therapies: evidence for action*. Geneva: World Health Organisation, 2003.
3. WHO. *Adherence to long-term therapies: evidence for action*. Geneva: WHO, 2003.
4. National Institute for H, Care E. Medicines adherence: involving patients in decisions about prescribed medicines and supporting adherence; 2009. http://www.nice.org.uk/guidance/cg76/resources/guidance-medicines-adherence-pdf2009
5. Horne R, Weinman J, Barber N, *et al*. *Concordance, adherence and compliance in medicine taking: a conceptual map and research priorities*. London: National Coordinating Centre for NHS Service Delivery and Organisation, 2005.
6. Charles C, Gafni A, Whelan T. Shared decision-making in the medical encounter: what does it mean? (or it takes at least two to tango). *Soc Sci Med* 1997;44:681–92.
7. Marinker M, Shaw J. Not to be taken as directed putting concordance for taking medicines into practice. *BMJ* 2003;326:348–9.
8. Barnett K, Mercer SW, Norbury M, *et al*. Epidemiology of multimorbidity and implications for health care, research, and medical education: a cross-sectional study. *The Lancet* 2012;380:37–43.
9. Watt G. The subversive challenges of multimorbidity. *Br J Gen Pract* 2017;67:280–1.
10. Armstrong N, Swinglehurst D. Understanding medical overuse: the case of problematic polypharmacy and the potential of ethnography. *Fam Pract* 2018;35:526–7.
11. Royal Pharmaceutical S. Medicines optimisation: helping patients to make the most of medicines, 2013. Available: http://www.rpharms.com/promoting-pharmacy-pdfs/helping-patients-make-the-most-of-their-medicines.pdf
12. National Institute for H, Care E. Medicines optimisation: the safe and effective use of medicines to enable the best possible outcomes, 2015. Available: https://www.nice.org.uk/guidance/ng5
13. Duerden M, Avery T, Payne R. *Polypharmacy and medicines optimisation: making it safe and sound*. London: The King's Fund, 2013.
14. Skrabanek P. Risk factor epidemiology: science or non-science. In: *Social affairs unit, health, lifestyle and environment: countering the panic*. London: Social Affairs Unit, 1991.
15. Heath I. Role of fear in overdiagnosis and overtreatment—an essay by Iona Heath. *BMJ* 2014;349.
16. O'Sullivan JW, Stevens S, Hobbs FR, *et al*. Temporal trends in use of tests in UK primary care, 2000-15: retrospective analysis of 250 million tests. *BMJ* 2018;363.
17. Swinglehurst D, Fudge N. The polypharmacy challenge: time for a new script? *Br J Gen Pract* 2017;67:388–9.
18. Sinnott C, Hugh SM, Boyce MB, *et al*. What to give the patient who has everything? A qualitative study of prescribing for multimorbidity in primary care. *Br J Gen Pract* 2015;65:e184–91.
19. Webber MM, Rittel H. Dilemmas in a general theory of planning. *Policy sciences* 1973;4:155–69.
20. Masnoon N, Shakib S, Kalisch-Ellett L, *et al*. What is polypharmacy? A systematic review of definitions. *BMC Geriatr* 2017;17:230.
21. Dixon-Woods M. What can ethnography do for quality and safety in health care? *Qual Saf Health Care* 2003;12:326–7.
22. McIntosh P. *Action research and reflective practice: creative and visual methods to facilitate reflection and learning*. Routledge, 2010.
23. Public Health England (PHE). National general practice profiles; 2019.
24. Stake RE. Qualitative Case Studies. In: Denzin NK, Lincoln YS, eds. *The SAGE Handbook of qualitative research*. 3rd Sage Publications, 2005: 443–66.
25. Swinglehurst DF N. APOLLO-MM: related projects, 2019. Available: http://www.polypharmacy.org.uk/related-projects/
26. Wengraf T. *Qualitative research interviewing: biographical narrative and semi-structured methods*. London, Thousand Oaks, New Delhi: Sage Publications, 2001.
27. Gaver B, Dunne T, Pacenti E. Design: cultural probes. *Interactions* 1999;6:21–9.
28. Boehner K, Gaver W, Boucher A. Probes. In: *Inventive methods*. Routledge, 2012: 199–215.
29. Wherton J, Sugarhood P, Procter R, *et al*. Designing assisted living technologies 'in the wild': preliminary experiences with cultural probe methodology. *BMC Med Res Methodol* 2012;12:188.
30. Greenhalgh T, Wherton J, Sugarhood P, *et al*. What matters to older people with assisted living needs? A phenomenological analysis of the use and non-use of telehealth and telecare. *Soc Sci Med* 2013;93:86–94.
31. Feldman MS, Pentland BT. Reconceptualizing organizational Routines as a source of flexibility and change. *Adm Sci Q* 2003;48:94–118.
32. Pentland BT, Feldman MS. Organizational routines as a unit of analysis. *Ind Corp Change* 2005;14:793–815.
33. Feldman MS. A performative perspective on stability and change in organizational routines. *Ind Corp Change* 2003;12:727–52.
34. Feldman MS. Organizational Routines as a source of continuous change. *Organ Sci* 2000;11:611–29.
35. Geertz C. Thick description: toward an interpretive theory of culture. In: *The interpretation of cultures*. New York: Basic Books, 1973: 3–30.
36. Swinglehurst D, Greenhalgh T, Russell J, *et al*. Receptionist input to quality and safety in repeat prescribing in UK general practice: ethnographic case study. *BMJ* 2011;343:d6788.
37. Dionysiou DD, Tsoukas H. Understanding the (Re)Creation of Routines from Within: A Symbolic Interactionist Perspective. *Acad Manage Rev* 2013;38:181–205.
38. Wright A. Organizational routines as embodied performatives: a communication as constitutive of organization perspective. *Organization* 2016;23:147–63.
39. Schoeneborn D, Kuhn TR, Kärreman D. The communicative constitution of organization, organizing, and organizationality. *Organ Stud* 2018.
40. Cooren F, Kuhn T, Cornelissen JP, *et al*. Communication, organizing and organization: an overview and introduction to the special issue. *Organ Stud* 2011;32:1149–70.
41. Ashcraft KL, Kuhn TR, Cooren F. 1 Constitutional Amendments: "Materializing" Organizational Communication. *Acad Manag Ann* 2009;3:1–64.
42. Putnam L, Nicotera AM. *Building theories of organization: the constitutive role of communication*. New York, Abingdon: Routledge, 2009.
43. Cooren F, Fairhurst GT. Dislocation and stabilization: how to scale up from interactions to organization. In: Putnam L, Nicotera AM, eds. *Building theories of organization: the constitutive role of communication*. New York, London: Routledge, 2009: 117–52p..

For peer review only - http://bmjopen.bmj.com/site/about/guidelines.xhtml

BMJ Open: first published as 10.1136/bmjopen-2019-031601 on 22 August 2019. Downloaded from http://bmjopen.bmj.com/ on August 23, 2019 by guest. Protected by copyright.

44. Boje DM. *Storytelling organizations*. SAGE, 2008.
45. Gabbay J, le May A. Evidence based guidelines or collectively constructed "mindlines?" Ethnographic study of knowledge management in primary care. BMJ 2004;329:1013–7.
46. Czarniawska B. A theory of organizing. In: Publishing EE, ed. *2nd*, 2014.
47. Iedema R. Creating safety by strengthening clinicians' capacity for reflexivity. BMJ Qual Saf 2011;20(Suppl 1):i83–6.
48. Iedema R, Mesman J, Carroll K. *Visualising health care practice improvement: innovation from within*. London, New York: Radcliffe Publishing Ltd, 2013.
49. Iedema R, Carroll K, Collier A, *et al*. *Video-reflexive ethnography in health research and healthcare improvement*. Boca Raton London New York: CRC Press, 2019.
50. Iedema R, Carroll K. Discourse research that intervenes in the quality and safety of care practices. Discourse & Communication 2010;4:68–86.
51. Schon DA. *The reflective practitioner: how professionals think in action*. New York: Basic Books Inc, 1983.
52. Bate P, Robert G. Experience-Based design: from redesigning the system around the patient to co-designing services with the patient. Qual Saf Health Care 2006;15:307–10.
53. Bate P, Robert G. *Bringing user experience to healthcare improvement: the concepts methods and practices of experience-based design*. Radcliffe Publishing, 2007.
54. Thomson A, Rivas C, Giovannoni G. Multiple sclerosis outpatient future groups: improving the quality of participant interaction and ideation tools within service improvement activities. BMC Health Serv Res 2015;15:105.
55. Nicolini D. *Practice theory, work, & organization: an introduction*. Oxford: Oxford University Press, 2012.
56. Nicolini D. Zooming in and zooming out: a package of method and theory to study work practice. In: Ybema S, Yanow D, Wels H, *et al*, eds. *Organizational ethnography*. London, Thousand Oaks, New Delhi, 2009: 120–38Sage Publicationsp.
57. May C, Montori VM, Mair FS. We need minimally disruptive medicine. BMJ 2009;339:b2803.
58. Mair FS, May CR. Thinking about the burden of treatment. BMJ 2014;349:g6680.

For peer review only

BMJ Open: first published as 10.1136/bmjopen-2019-031601 on 22 August 2019. Downloaded from http://bmjopen.bmj.com/ on August 23, 2019 by guest. Protected by copyright.

For peer review only - http://bmjopen.bmj.com/site/about/guidelines.xhtml

BMJ Open

Organising polypharmacy: unpacking medicines, unpacking meanings – an ethnographic study

Journal:	*BMJ Open*
Manuscript ID	bmjopen-2021-049218.R1
Article Type:	Original research
Date Submitted by the Author:	12-Jul-2021
Complete List of Authors:	Swinglehurst, Deborah; Queen Mary University of London Barts and The London School of Medicine and Dentistry, Institute of Population Health Sciences<br>Fudge, Nina ; Queen Mary University of London, Institute of Population Health Sciences
**Primary Subject Heading**:	Qualitative research
Secondary Subject Heading:	General practice / Family practice, Patient-centred medicine
Keywords:	QUALITATIVE RESEARCH, PRIMARY CARE, THERAPEUTICS

SCHOLARONE™
Manuscripts

I, the Submitting Author has the right to grant and does grant on behalf of all authors of the Work (as defined in the below author licence), an exclusive licence and/or a non-exclusive licence for contributions from authors who are: i) UK Crown employees; ii) where BMJ has agreed a CC-BY licence shall apply, and/or iii) in accordance with the terms applicable for US Federal Government officers or employees acting as part of their official duties; on a worldwide, perpetual, irrevocable, royalty-free basis to BMJ Publishing Group Ltd ("BMJ") its licensees and where the relevant Journal is co-owned by BMJ to the co-owners of the Journal, to publish the Work in this journal and any other BMJ products and to exploit all rights, as set out in our licence.

The Submitting Author accepts and understands that any supply made under these terms is made by BMJ to the Submitting Author unless you are acting as an employee on behalf of your employer or a postgraduate student of an affiliated institution which is paying any applicable article publishing charge ("APC") for Open Access articles. Where the Submitting Author wishes to make the Work available on an Open Access basis (and intends to pay the relevant APC), the terms of reuse of such Open Access shall be governed by a Creative Commons licence – details of these licences and which Creative Commons licence will apply to this Work are set out in our licence referred to above.

Other than as permitted in any relevant BMJ Author's Self Archiving Policies, I confirm this Work has not been accepted for publication elsewhere, is not being considered for publication elsewhere and does not duplicate material already published. I confirm all authors consent to publication of this Work and authorise the granting of this licence.

For peer review only

Title: Organising polypharmacy: unpacking medicines, unpacking meanings – an ethnographic study

Authors

Deborah Swinglehurst MBBS, MA, MSc, PhD, FHEA, FRCGP
Institute of Population Health Sciences
Barts and The London School of Medicine and Dentistry
Queen Mary University of London
London, UK

Nina Fudge BA (Hons), MSc, PhD, FHEA
Institute of Population Health Sciences
Barts and The London School of Medicine and Dentistry
Queen Mary University of London
London, UK

Corresponding author:

Deborah Swinglehurst
Institute of Population Health Sciences
Barts and The London School of Medicine and Dentistry
Queen Mary University of London
58 Turner Street
London E1 2AB
UK

Email: d.swinglehurst@qmul.ac.uk

Word count: 4197 (excluding title page, abstract, article summary, references, and tables)

For peer review only - http://bmjopen.bmj.com/site/about/guidelines.xhtml

ABSTRACT

Objectives: We explore how older patients affected by polypharmacy manage the ‘hidden work’ of organising their medicines, how they make sense of this work and integrate it into their lives.

Design and setting: Ethnographic study observing patients over 18-24 months in patients’ homes, general practice and community pharmacy, in England, UK.

Participants and methods: Ethnographic case study including longitudinal follow-up of 24 patients aged 65 or older and prescribed ten or more items of medication. Our dataset includes: 562 hours of ethnographic observation across patients homes, community pharmacies and general practices; 47 audio-recorded interviews with patients about their lives and medicines practices; cultural probes (photographs, body maps, diaries and imagined ‘wishful thinking’ conversations); fieldnotes from regular home visits; telephone calls, and observation/video-recording of healthcare encounters. We apply a ‘practice theory’ lens to our analysis, illuminating what is being accomplished, why and by whom.

Results: All patients had developed strategies and routines for organising medicines into their lives, negotiating medicine-taking to enable acceptable adherence and make their medicines manageable. Strategies adopted by patients often involved the use of ‘do-it-yourself’ dosette boxes. This required careful ‘organising’ work similar to that done by pharmacy staff preparing multi-compartment compliance aids (MCCAs). Patients incorporated a range of approaches to manage supplies and flex their regimens to align with personal values and priorities. Practices of organising medicines are effortful, creative and often highly collaborative. Patients strive for adherence, but their organisational efforts privilege ‘living with medicines’ over taking medicines strictly ‘as prescribed’.

Conclusions

Polypharmacy demands careful organising. The burden of organising polypharmacy always falls somewhere, whether undertaken by pharmacists as they prepare MCCAs or by patients at home. Greater appreciation amongst prescribers of the nature and complexity of this work may provide a useful point of departure for tackling the key issue that sustains it: polypharmacy.

For peer review only - http://bmjopen.bmj.com/site/about/guidelines.xhtml

Strengths and limitations of this study

- Gathers rich qualitative data from a range of community settings and is grounded in the everyday experiences of participants
- Reveals decision-making and practices around medication management which are often hidden from view and unknown to professionals who prescribe and dispense medicines
- Uses innovative qualitative methods to explore aspects of everyday life which might be difficult to reach using more conventional approaches
- Prioritises depth of analysis over breadth, enabling richness of conceptual insights but limiting generalisability
- Patients' willingness to take part in our research might reflect their commitment to their medicines

Keywords

Polypharmacy; Multimorbidity; Qualitative Research; Primary Health Care

For peer review only

INTRODUCTION

Polypharmacy is one of three key action areas in the World Health Organization Global Challenge ‘Medication without Harm’.[1] The prevalence of polypharmacy is increasing. In one Scottish study 20% of adults were dispensed five or more drugs and 6% of adults were dispensed ten or more drugs.[2] Patients receiving ten or more drugs are at particular risk of harm, so-called ‘high risk’ polypharmacy.[3] Guthrie’s study showed a tripling in the prevalence of this phenomenon between 1995 and 2010.[2-5] Taking medication reliably and safely is demanding for patients, especially when complex medication regimens are involved.[6 7] Supporting patients in taking their medication is a key policy and professional commitment, enshrined within ‘medicines optimisation’: a “*person-centred approach to safe and effective medicines use, to ensure people obtain the best possible outcomes from their medicines*’.[3 8 9] Medicines optimisation is situated within an objective ‘rational use of drugs’ paradigm, which emphasises judicious prescribing by well-informed professionals of well-studied drugs to well-informed patients for well-defined conditions.[10] Achieving medicines optimisation is assumed to hinge centrally on a process of ‘shared decision making’ within the clinical consultation. This is emphasised in the NHS England 2020/21 update to the GP contract which requires Primary Care Networks to invite patients who are prescribed ten or more medications for a Structured Medication Review (SMR) with an appropriately trained clinical pharmacist or general practitioner.[11]

Notwithstanding the importance of medication reviews, most decision-making about medicines taking, and almost all of patients’ medication practices,[12] take place beyond the clinic, usually in patients’ homes. In the clinic, medicines are typically conceptualised as biomedical technologies (a disease, or risk of disease, is identified and a medicine is prescribed to ameliorate symptoms or reduce risk). But medicines can also be conceptualised as socially embedded phenomena, as representations that carry meanings and shape social relations.[13] Medicines are material *things* of therapy which have a social life,[14] and this becomes especially salient when medicines enter patients’ homes and daily lives. Here, patients construct a ‘lay pharmacology’ of personally relevant notions of safety, side effects and efficacy which both converge with, and diverge from, conventional biomedical perspectives.[15] Medication practices become sites of moral evaluations of self, society and

For peer review only - http://bmjopen.bmj.com/site/about/guidelines.xhtml

pharmaceuticals; peoples' relationship with medicines offers insight into their identities, and into these moral stories of self and society.[16] Consumption of medicines becomes a matter of embodied, purposeful, situated accomplishment,[7 17] involving a particular kind of *work* or expertise as patients devise ways to incorporate their medicines into their daily lives. Patients have to 'do' medicines adherence.[7]

Under this view, taking medicines is not only a matter of cognition and decision-making (shared or otherwise). It is also a very practical matter of *organising*, of managing medicines *into* daily life and establishing routines. Indeed, one of the key motivations for establishing routines is to *minimise* the cognitive demands involved in carrying them through. A balance must be struck. As medicines routines become more reliable (and the attention invested in them reduces), the unintentional risk of missing doses increases, so patients must devise further strategies to anticipate this possibility.[18 19] Huyard et al. studied unintentional non-adherence amongst patients prescribed single items of long term medication and found that patients used a range of strategies to support their adherence.[19] These included *anchoring* (scheduling pill-taking with another pre-existing routine), *spatial positioning* of pills in ways that make pill-taking more reliable, and methods for *verifying* that pill-taking has occurred.[19]

When polypharmacy involves large numbers of drugs it presents particular challenges to both professionals and patients. If a genuinely *person-centred* approach to medicines optimisation is sought, research is needed which illuminates these challenges, by attending to professionals' and patients' subjective, context-specific evaluations of medicines, their lived experiences and everyday practices. When patients devise routines to support their medicines-taking they are invested in biographical work, in what Huyard describes as the '*microintegration of their illness deep into patients' everyday lives*'.[19] Our research adopts an ethnographic approach to explore these practices. Ethnography is well suited to investigating complex multifaceted phenomena by making tacit knowledge and practices visible.[20] It involves immersion in the field, an attitude of 'appreciation' towards the social world *as it is*, presentation of 'first hand' accounts of observations, and acceptance that research is an active process in which accounts of the world are constructed through selective observation and theoretical interpretation.[21]

For peer review only - http://bmjopen.bmj.com/site/about/guidelines.xhtml

In this paper, we focus primarily on the practices of older adults who live at home and are prescribed ten or more separate items of medication. To set the scene, we begin in a community pharmacy, with a brief ethnographic description of practices we observed as professionals prepared multi-compartment compliance aids (MCCAs) or 'dosette' boxes for some patients affected by polypharmacy. This draws attention to the organisation of polypharmacy as a form of 'work'. We then shift our ethnographic gaze from the professional context into the private context of patients' homes to address the question: '*How do patients organise their medicines and medicine-taking?*' We explore the meanings embedded in these everyday acts of organising and illuminate who is doing the work and what this work involves. We show how patients draw on personal, social, material and hermeneutical resources in their efforts to adhere to their medicines within the context of their daily lives, and how their lives are shaped by, and in turn shape, their medicines regimens.

METHODS

The study reported here is part of the wider APOLLO-MM study (Addressing the Polypharmacy Challenge in Older People with Multimorbidity) The methods have been described in detail elsewhere.[22] Table 1 summarises our data collection across general practices, community pharmacies and patients' homes. Our approach has enabled us to build a rich picture of our patient participants' experiences in both personal and professional contexts. We have used O'Brien et al's Standards for Reporting Qualitative Research (SRQR).[23]

Table 1. Summary of data collection methods

**Method/approach**	**Details of data collection**
Organisational ethnography of GP practices	184 hours observation in three GP practices, observing professional practices relevant to prescribing including shadowing staff and 6 interviews
Organisational ethnography of community pharmacies	140 hours in four community pharmacies, observing professional practices relevant to

For peer review only - http://bmjopen.bmj.com/site/about/guidelines.xhtml

For peer review only

	polypharmacy, including shadowing staff and 21 interviews[24]
Longitudinal follow up of 24 patients	Ethnographic observation in patients' homes (66 home visits) and regular follow up conversations by telephone (258 telephone calls). We accompanied participants to selected hospital and GP consultations and on trips to the local pharmacy (clinical consultations = 29 of which 18 were video recorded).
Biographical narrative interviews with patients	24 in-depth narrative interviews using Wengraf's biographic-narrative-interpretive method,[25] eliciting a narrative with one opening question ("please tell me the story of your life since you were first advised to take medicines") and using the participant's account to elicit further narratives.
In-depth interviews with patients about their medicines	23 in-depth interviews focused on participants' medicines and medicines practices (one participant withdrew from study due to a decline in health before this interview could be completed).
Cultural probes with 14 of the patient participants.	Cultural probes encouraged participants to depict their lives with their medicines through a number of optional activities:[26]<br>• giving participants a camera and asking them to take pictures of what their medicines mean to them<br>• drawing body maps of how they are feeling and how their medicines make them feel<br>• completing diaries of social contact

For peer review only - http://bmjopen.bmj.com/site/about/guidelines.xhtml

	• 'wishful thinking': participants imagined conversations they would like to have with a healthcare professional about their medicines

Setting and data collection

We recruited a purposive sample of 24 patients, 14 from two inner city general practices in an area of high deprivation, 10 from a suburban general practice in a relatively prosperous city. Patients were aged 65 or over, living at home, and were prescribed 10 or more regular items of medication which were dispensed by our study pharmacies. The sample reflects diversity of age (65-94); gender (11 men, 13 women); socioeconomic status; number of items of regular medication; co-morbidities. We excluded patients unable to speak adequate English. All patients gave written informed consent to participate in the study. Our sampling was guided by Malterud's notion of information power and reflects our detailed data collection methods and multiple opportunities to interact with study participants.[27] Data was collected between September 2017 and July 2020.

Each patient constituted a 'case'. We followed our participants' lives and experiences over an 18-24 month period of ethnographic observation involving a range of data collection activities (see Table 1).

All interviews took place in participants' homes and were audio-recorded. Two researchers (NF, a social anthropologist and DS, an academic GP, both experienced ethnographers in healthcare settings) undertook data collection and analysis (author initials in the text refer to which researcher conducted interviews or documented field notes). Two patients withdrew from the study after 8 and 11 months of follow-up.

Analysis

Fieldnotes and interview transcripts were shared between the researchers and we maintained a shared digital reflexive journal using Evernote® to document analytic

For peer review only - http://bmjopen.bmj.com/site/about/guidelines.xhtml

memos and ongoing analytic insights, in addition to regular data analysis meetings. We used QSR NVivo 12 qualitative data analysis software to manage the data,[28] and adopted an interpretive approach to analysis. Our analysis is informed by practice theory[29 30] which conceptualises practices as arrays of embodied human activity and draws attention to assemblages of people, technologies, artefacts and their interconnections in context, including shared practical understandings, 'know-how', skills, tacit understandings and dispositions. All names reported in this paper are pseudonyms, and case narratives have been adapted to assure anonymity of research participants.

Patient and Public Involvement

We have an online patient panel of five members and a project advisory group with 11 members: lay chair; academics; health professionals; representation from Age UK; two patient members. Patients were involved in: proposal development; designing participant materials/project website (www.polypharmacy.org.uk); application for ethical approval; project launch event; piloting interviews; study design and conduct.

STUDY FINDINGS

Medications are much more than material objects with physiological effects; they are also representations that carry meanings and shape social relations as they evolve in conjunction with individuals and collectivities.[10]

We begin by presenting an ethnographic account of the professional work of organising polypharmacy. This account focusses on the preparation of multi-compartment compliance aids (MCCAs or 'dosettes'). These were dispensed to four of our 24 research participants at the outset of our study; six participants received them by the end of our ethnographic follow up. MCCAs are typically dispensed to patients affected by polypharmacy who are having difficulty managing their medicines independently. In our observations of the 'backstage' areas of community pharmacies we were struck by the scale and complexity of this work.[31] Table 2 presents a brief description based on one community pharmacy site (for a more

For peer review only - http://bmjopen.bmj.com/site/about/guidelines.xhtml

detailed account of our observations in community pharmacy see Fudge and Swinglehurst 2021).[24]

Table 2. Ethnographic account of MCCA/dosette preparation in Willow Pharmacy

Willow Pharmacy is the ‘dosette hub’ for the Woodland Independent Pharmacy group. The work of preparing dosettes is called ‘*being on production’* and is a technology-supported routine involving a full-time dosette robot and its human co-workers, typically three pharmacy technicians, also working full-time to ensure supply keeps up with demand. The robot cannot work unsupervised and needs constant attention. Demand for dosettes is increasing every month, as displayed on a hand-written poster on the wall in the ‘*production area*’ where a running tally is kept. The pharmacy technicians are engaged in a constant, well-coordinated round of stocktaking, ‘*de-blistering*’ drugs, ‘*replenishing*’ the robot and running each production session. De-blistering is the process by which the staff prepare the medicines for the robot, as it cannot process medicines from their original packs. Pills are ‘*popped*’ out, one by one, from their blister packaging into containers which slide into the robot. Some staff use a de-blistering machine for this, pulling a large metal lever to press the pills out of their packets into interim containers for checking by the pharmacist prior to replenishment. Others do it manually. Technicians work at speed and use a wide range of technologies including: computer programmes; Excel spreadsheets, often printed out; written log books; printed cards; scribbled post-it notes. All contribute to maintaining the smooth flow of routines and a sense of predictability as they generate dosettes in 4-weekly batches ready to stack on the shelves awaiting final checking. Medicines are referred to as ‘*fast lines*’ (i.e. there is a lot of demand for them by the pharmacy’s clients) or ‘*slow lines*’ (i.e. less demand). This categorisation is an important component of decision-making regarding whether a particular medicine warrants inclusion in the robot. ‘*Slow lines*’ may be designated ‘*externals*’ (meaning that the medicine is added to a dosette box by hand once the robot has done its job). The integrity of medicines may be at risk if they are left out of their packaging for too long. Expensive lines are also dealt with as ‘*externals*’ so that the pharmacy can remain agile in its response to market price fluctuations. Despite everyone’s best efforts to rationalise production, there are inevitably plenty of exceptions and contingencies to deal with. We often see filled dosettes recovered again from the shelves as staff attend to unanticipated changes in patients’ prescriptions. This entails a painstaking process of using tweezers as a tool to isolate, break off and remove the backing from relevant cells of the dosette, then carefully removing and/or adding the relevant medicines, sealing the cell up again and attending to all the associated paperwork and electronic documentation. If the technician in the ‘*dosette corner*’ receives too many requests for changes of this kind to the contents of dosettes in quick succession, the job quickly becomes very stressful.

This account offers some insight into the scale and complexity of the organisational work that polypharmacy presents and the range of professional considerations that are brought to bear as routines are developed to ensure that work proceeds as swiftly and safely as possible. It contextualises polypharmacy as the locus of *work.* In the remainder of this paper we consider the organisational work of our patient participants. We focus on patients who were dispensed all of their medications in their original packaging, where the burden of organisational work sits squarely with

For peer review only - http://bmjopen.bmj.com/site/about/guidelines.xhtml

the patient. At the outset of our study 20 of the 24 participants received all their medication in original packaging.

Many participants expressed a sense of obligation and resignation with regards to taking medicines ‘as prescribed’, often expressing medicine-taking as a non-negotiable aspect of their lives. At the same time, they readily adapted their regimens to make them manageable. Their organisational efforts reflect a balance of these two broad commitments.

> *I have to take them, that’s is. I have to accept it…I mean if I was on less, yeah, I’d be happy, but I’m not, so I just have to take them and that’s it*

(Biographical interview, Marco, DS)

> *I’m supposed to take that [carbocisteine] two tablets three times a day. I don’t. Morning and evening. I usually forget the afternoon one, and it’s possibly insufficient, but I have to balance one inconvenience with another inconvenience.*
>
> And later he goes on:
>
> *It [taking medicines] always takes place after breakfast, although breakfast can be anything between eight and ten. I know that I should be taking them [the ‘morning’ tablets] at a specific time each day in preference, but in life it doesn’t always happen like that.*

(Medicines interview, Charles, DS)

Participants invested considerable time and effort into organising their medicines into their lives, motivated by their striving to adhere to their medicines regimens:

> *I’m actually very methodical about it and I almost never miss any, which I believe is quite unusual, but it’s because I’ve got this routine that I feel comfortable…it’s been going on exactly the same for a number of years.*

(Medicines interview, Elaine, NF)

For peer review only - http://bmjopen.bmj.com/site/about/guidelines.xhtml

Everything's in the kitchen…when they're on top of the microwave, I can't forget them.

(Medicines interview, Maria, NF)

I try to be organised, because it's easier to do that than getting stuff out every day. I'd find that very confusing.

(Medicines interview, Marian, DS)

When I ask Charles if he will show me his medicines he gets up quickly from his chair and moves towards the kitchen. I don't feel able to follow him there. He reappears with a little tray with a lip round the edge about an inch high. In it there is a very neatly organised set of medicine boxes lined up, with some sachets of Laxido [a laxative] propped up along one side. It looks very orderly and I wondered how he managed to find a tray that is such a perfect fit for his medicines boxes, or indeed what would happen if he were to stop half of them as they wouldn't tessellate and hold their place quite as they do in this neat arrangement. There is one open strip of capsules adjacent to the Laxido sachet, but otherwise everything is kept in its original box. He tells me that they are kept in a kitchen cupboard and later when I switch on my audio-recorder for an interview he expands: "When we have guests or family or something, I'm told to put the bloody things away and not to display them and show off, and that sort of thing". Charles grimaces as he explains that his wife, a retired GP, does not like his medicines to be visible. He goes on to say that his wife thinks they should be kept discretely out of view. As he talks me through his medicines he lifts up the little tray from the coffee table onto his lap and goes through them meticulously, one by one, though he is careful to tell me that he is approaching them in 'no particular order'.

(Fieldnotes, home visit to Charles, DS)

These quotes and ethnographic notes point not only to the ways in which space, time and physical arrangements are harnessed to routinise and support medicines-taking but also how efforts at organising are a considered social performance. Our participants varied in the extent to which their medical complaints were made visible

For peer review only - http://bmjopen.bmj.com/site/about/guidelines.xhtml

through their medicines to others who might visit the home. This finding resonates with the work of Palen and Aalokke who studied medicines management amongst elders in assisted living apartments in Denmark.[32] A key point of difference is that in the Danish study the participants received at least daily visits from a mobile health care worker who collaborated with elders in their medicines management. In our study home visits by health professionals were rare, even for our six housebound patients. Patients devised their own strategies and routines, although this often involved considerable resourcefulness and collaboration with others.

Given the number of items (10 – 30) and variety (tablets, creams, eye drops, injectables, inhalers and inhaled oxygen) of medicines prescribed to participants in our study their medicines regimens were inevitably very complex and needed careful organising. For example, one study participant consumed 21 tablets every morning, 15 tablets every evening and various 'as required' medicines in between; seven of our participants were prescribed 15 or more different items of daily medication. Despite the complexity of this work it was difficult to persuade our participants that there may be value in us learning about it as researchers. Whilst it was clear to us that they had given much thought to how to integrate their medicines into their lives, they mostly regarded their medicines as uninteresting, routine, normal, mundane day-to-day business, but nevertheless '*a nuisance*'. Unlike their medical histories, which they often recounted in detail and in a well-rehearsed manner, they often struggled to articulate their approaches to organising and consuming their medicines ('*I just take them!*'). In part this may reflect the extent to which their routines had become embodied and successfully integrated into daily life; they no longer required ongoing careful consideration.

We now present three 'telling cases',[33] vignettes distilled from detailed longitudinal narrative case studies of each study participant. We seek to strike a balance between providing sufficient rich detail to weave together our analytic insights, whilst being succinct enough to convey the breadth of experience and practices across different patient participants. We draw primarily on our interview data and ethnographic field notes, with brief reference to some of our other data collection activities. Our objective in our selection of data for presentation, and in our approach to presenting this data as vignettes is to illuminate patients' hidden work of organising medicines into their lives in their efforts to adhere to complex prescribed

For peer review only - http://bmjopen.bmj.com/site/about/guidelines.xhtml

medication regimens. These cases are not selected on the grounds of typicality but for their capacity to situate polypharmacy within a social context and challenge normative biomedical conceptualisations of polypharmacy. They speak to new ways of knowing the phenomenon of polypharmacy as it shapes the lives of patients experiencing complex multimorbidities. See Table 3 (Marco); Table 4 (Jackie) and Table 5 (Zac)

For peer review only

For peer review only - http://bmjopen.bmj.com/site/about/guidelines.xhtml

Table 3. Vignette of Marco

Marco is a retired chef. His narrative begins over 10 years ago at age 60 when he was diagnosed with diabetes and then with a '*cardiac problem*' which led to him having major surgery. He lives in an owner-occupied flat on the top floor of a low rise block in a deprived inner city area. We usually sit outside on his covered balcony when I visit; he smokes cigarettes and sips '*real*' coffee ('*life's little luxuries'*) as he talks.<br>He tells me about his blackouts, painful joints, lack of youthful energy and strength, fingers which get sore from finger prick tests (which he tactfully avoids except in the week before his diabetes check-up), and his warfarin levels which '*go up and down like a bloody yo-yo*'. He has no upper teeth and his dentures don't fit as he is awaiting dental treatment, so despite his fondness for cooking, eating is difficult.<br>Marco displays a sense of resignation about his ill health ('*I don't bother the doctor with everything – what can they do?'*). He is prescribed 15 items of medication but has not seen his GP for three years, although he attends the surgery regularly for nurse-led diabetes checks and warfarin monitoring blood tests ('*a nuisance'*). His medicines mean '*survival…I have to take them for life to keep me alive'* but at the same time they '*get on my nerves'* and '*I wouldn't take any of them by choice…but I don't have any choice*.'<br>Marco's pharmacist offered to package his medicines into a MCCA and deliver them direct to his flat, but he declined:<br><br>'*Sometimes you're offered a service but it doesn't mean you really need that service, you know what I mean? I can do it myself, it gives me a little exercise…it can wait a little while before I need the service…I can do it myself now*.'<br><br>He continues:<br><br>'*There's a couple of people in this block I noticed they have it [their medicines] delivered; they have this delivered, that delivered, when they're capable and it would be good for them to go around there and get it, you know?'* He goes on: *'sometimes it's just laziness.'*<br><br>I soon learn that Marco's friend Vicky helps him organise his medicines, though he tells me repeatedly that he could easily manage himself. Vicky is in her 40's and visits every day; she sometimes answers the phone when I call. She prepares his Do-It-Yourself [DIY] cassette-type MCCA on Sundays at a frequency that depends on the results of Marco's warfarin monitoring [International Normalised Ratio or INR]. If his INR is stable she may prepare four weeks at once. After all, Marco says '*you don't want to be undoing it all again do you? It would be nuisance'*. Vicky also prompts him to order his medication from his GP using an online service when supplies are low. Marco says he knows '*roughly*' what his tablets are, although a video-recording of one of his clinical consultations with his GP casts some doubt on how well he knows them by either name or purpose. Warfarin and insulin are the only medications he ever refers to by name – the others he knows by condition (diabetes, blood pressure), shape and size (the '*big one'*), time of day (the '*night one*', or organ ('*heart*'). This knowledge is adequate for his organisational purposes.<br><br>Marco explains that the DIY MCCA he uses currently is bigger than previous models he has tried - his medicines outgrew the smaller versions. The cassette consists of a stack of seven rectangular boxes labelled by day of the week. He takes the 'Monday' box from the bottom, causing the others to slide downwards within the supporting rack. There are 12 pills distributed across the four cells but each day he shifts the 'midday' ones that Vicky has prepared into the 'morning' cell (any time from 10 am until 1pm, it turns out) and shifts the 'teatime' ones into the 'evening' cell; two rounds of medicine-taking is easier than four. It seems that Vicky feels obliged to organise them as the prescription dictates, leaving Marco free to take responsibility for his personal re-organisation. He tries to take his evening medicines 20 minutes before eating but if he forgets - which he admits he

For peer review only - http://bmjopen.bmj.com/site/about/guidelines.xhtml

sometimes does - he takes them two hours after eating '*or thereabouts*'. He finds it '*very, very annoying'* that his medicines change in shape and colour so often, especially as he is colour blind and colours are confusing at best of times. His insulin pen sits on the coffee table, with extra supplies in the fridge. He keeps three inhalers in a 'Man Tin'. There is a glyceryl trinitrate (GTN) spray by the bed '*just in case my heart starts messing around*' and he adds '*it comes out with me all the time*'. In addition he keeps a basket in the bathroom where:

'*I do accumulate a little surplus, but I try not to, not so much, you know, just a little bit so it gives me an extra three or four days, besides what they give me, you know?.. But I don't have a massive supply, because what happens is if she [the GP] changes one of them in strength and they won't take the tablets back or anything back… only a few, not stocking up, you know.*'

He retrieves his warfarin paperwork which is tucked behind an ornament on the mantelpiece. He points to the date of his next appointment in one weeks' time. He explains he has to take his '*yellow book'* to the surgery every time he needs a prescription for warfarin; this requirement doesn't align well with the electronic routines now in place for ordering medicines online and transferring prescriptions between his GP and pharmacist.

On a later visit, Marco shares a poignant story about Vicky. When he met her a few years ago she was unable to manage her money, was overspending and not caring for herself. Marco bought her a tin, with two compartments – one for '*this week's*' money and one for '*next week's*' money – a kind of 'financial compliance aid' and has helped her '*get on her feet'* financially. Marco told me that he had advised Vicky that '*it is easy to live a life in which you just don't care about anything. It is harder to live a life in which you care.'*

For peer review only - http://bmjopen.bmj.com/site/about/guidelines.xhtml

Table 4. Vignette of Jackie

Jackie was diagnosed with a lifelong neurological condition as a teenager when she spent a year in hospital unable to walk. Her complex biography includes a stillbirth, a prolonged episode of severe loss of vision from which she recovered, numerous operations, diabetes, domestic abuse, two marriages. Now in her 70's she has ongoing chronic pain.<br><br>Jackie lives in social housing with her son and is prescribed 11 items of regular medication. She begins our interview by dropping her medicines accidentally on the floor. It is not unusual for us to find pills on the carpets when we visit our research participants; they are often clearly beyond their reach. With some difficulty Jackie manages to retrieve them and explains '*I take a metformin and something that goes with the metformin beginning with an 'A' – it's on my list somewhere – they go together in the morning*'. She refers to the '*thing that goes with the metformin*' four times during our interview and I conclude this is how she 'knows' and remembers it, though I later learn it is also '*the orange thing*'. She tells me '*I like to get them down my throat as quick as I can, because some of them have got some horrible taste to them…powdery, at the back of your throat, they lay there*'. She explains that the bad taste stays for about an hour. She goes on: '*In the evening, I take a metformin after a meal, on its own, and then - about no later than 10 o'clock - I take a co-codamol and carbamazepine and something beginning with 'E' which is a cholesterol tablet. And then just before I go to bed I take my diazepam to stop me shaking in the night*'. Through the day she is also guided by her pain, adjusting her medications '*according to how the pain is*' and she has other medicines which she takes to reduce the '*acid*' – a symptom she attributes to '*taking so many of them* [medicines]'. And then there are the '*terrible'* eye drops which she cannot manage independently because of the effects of her neurological condition on her hands:<br><br>*'I can hardly get them into my eyes. It's terrible. My son has to do it, because whenever anyone comes near my eye I shut it. And I said 'how long's that for? and she [the GP] said 'for life'.*<br><br>She thinks she is supposed to have her eye drops four times a day but as her son goes out to work at 06:45 there is no one to help with them during the day. A neighbour has offered but Jackie doesn't want to '*keep bothering people*'.<br><br>Jackie has a faded, well-used, Do-It-Yourself MCCA - one compartment per day, the days of the week now barely visible on the lid. David, her son, fills it every Sunday. She explains that she has got it out for me to see, but usually it's kept '*out of the way*' in the kitchen. Each compartment contains eight tablets (five separate items of medication). Jackie selects her tablets from each single compartment four times per day, choosing them by shape and colour. She is unsure of some of their names but, like Marco, she recognises them by their material properties. She is more than capable of filling this box herself, but David takes pride in helping with this crucial technical aspect of Jackie's care and she is grateful that '*I've got somebody keeping an eye on me*'. His role in helping her with her medicines management affirms his responsibility towards her as her official carer, alongside the more personal, hands-on aspects of her care, such as turning her most nights when she calls for him as she gets stiffness and spasms in her legs.<br><br>Although it is fiddly, Jackie manages to empty her 'as required' medicines (she calls them '*her spare ones*') from their blister packaging herself, and puts them together in a separate small '*spare tin*': co-codamol for pain, prochlorperazine for dizziness and omeprazole for indigestion (a pill which degrades outside its original packing). Like her regular medication in her DIY MCCA the different '*spares*' are mixed together. When we visit, this tin is in the kitchen, but it accompanies her to bed at night and fits neatly in her handbag for trips outdoors.

For peer review only - http://bmjopen.bmj.com/site/about/guidelines.xhtml

In addition, Jackie has a basket under her bed where she keeps tablets in their original packs. This is where she places medicines when they are delivered to her house by the pharmacy, and she tries to anticipate her needs by staying about one month ahead. She monitors her basket daily when she gets up and when she goes to bed, and calls the pharmacist on her mobile phone direct from her bed when she recognises her supplies are low. By keeping them under her bed and checking them daily she knows she won't '*get to a stage, I'd think Oh My God, I've got no tablets*'. She tries to strike a compromise between running out of medication and falling foul of the rather stark warning on the repeat prescription list she has from her doctor's surgery which reads: '*Don't stock-pile medicine at home – only order what you need*'. Like her MCCA, she keeps these medicines '*out of my way all day because they are upstairs*'. She prefers her medicines to be out of sight when she is not dealing directly with them.

Jackie speaks fondly of her local pharmacy and says they '*know me, because they call me Jackie, so they know me really well*'. But she complains that '*they* [the medicines] *never all run out together. It's a nightmare. I feel as though I am permanently ringing up the chemist for my tablets*'. We ask Jackie if there is one thing she could change about her medicines what would this be. Without hesitation she replies '*Not take any of them!*'

For peer review only

For peer review only - http://bmjopen.bmj.com/site/about/guidelines.xhtml

Table 5. Vignette of Zac

Zac, who is almost 90 is a retired secondary school teacher. He has lots of friends, is active in his church and serves his local community by running errands and cooking for people frailer than he. He tells me he derives great satisfaction from helping others. He adopts a dutiful, independent approach to his medicines and often talks about being a 'good' patient – '*my doctor tells me that I'm one of her best patients because I seem to do the right things, eat the right things and take the medication'*. He tells me that people cannot believe he is the age he is: *'It is my education and my faith that have got me through…and I look after myself*'.<br><br>He keeps a three-month supply of medicines in a carrier bag by his armchair in his living room. Every Saturday, without fail, he organises his medicines for the following week. He lines up his morning and evening tablets, and puts them into 14 separate small plastic bags, wrapped inside tissue paper. He then puts them into his trouser pockets: left pocket for morning tablets and right pocket for evening tablets. He keeps a whole week's supply here, taking a packet from each pocket every morning and evening. The system works well for Zac. His medicines are high priority, kept close to his person and his busy lifestyle need not interrupt his medicines-taking - nor vice versa; he can keep religiously to his schedule wherever he happens to be.<br><br>Like Marco, Zac has been offered a pharmacy-prepared MCCA and has resisted ('*as long as I have my faculties*'). He heard on the news that people make mistakes with the doses of medication in these boxes; he doesn't want anyone '*mixing things up'* and his '*brain is turning over well*'.<br><br>A few months later, Zac is admitted to hospital. Some of his diabetes pills are stopped, he is advised to start insulin injections and he now receives a weekly MCCA. No one discussed the MCCA with him and he is unhappy about it, protesting '*while I am capable I want to do it myself'*. He now feels '*on edge'* every Wednesday and stays in his flat waiting for his medicines to drop through the letterbox. On one occasion they did not arrive until after 7pm, after his pharmacy's advertised closing time and he became extremely worried. On another, he noticed the '*cholesterol medicine*' was missing. In our cultural probe activity ('wishful thinking') he explains that he wishes he could have a conversation with his GP about going back to his old system, adding '*I want to be in control of my own destiny'*. He wants to be free to go out and dislikes the weekly round of anxiety that he may run out of pills. He walked to the surgery several times hoping to secure an appointment to discuss this, only to be told there were no appointments.<br><br>Several months passed, before we observed a clinical consultation between Zac and his GP. Zac explained '*I'm not very happy with the dosette box… they restrict me in my work and my movement.*' The GP explained that Zac could have more than one box delivered at a time and promptly arranged this with the practice administrator responsible for MCCAs. Zac continued cautiously: '*I'm not particularly keen on dosette boxes. I'm quite careful about medicines'* but the GP did not get to the bottom of Zac's concern.<br><br>In a conversation after the appointment, Zac explained to us that pharmacists can make mistakes with the medicines '*so you still have to check that they've done it properly'*, suggesting that he did not feel relieved of the organisational burden but had lost the sense of control and freedom that he enjoyed before. He complained that sometimes he can't recognise the tablets now because the brands change and went on to say that people who like dosettes are 't*aking the easy route*'.<br><br>We realise his diligence is being undermined and he speculates '*maybe they thought now I'm over 80 I can't manage*?' He tells us he will try out the '*four weekly dosettes*' then go

For peer review only - http://bmjopen.bmj.com/site/about/guidelines.xhtml

back to the GP again and ask '*why I even need to be on them [the dosettes]*'. Several months later in our final discussion with Zac, before the end of our data collection period, he had yet to have this conversation with his GP.

These three cases convey a 'thick description' [34] of the nature and complexity of our study participants' medicines practices. Drawing on these cases as exemplars from our wider dataset we now synthesise these accounts to identify some key areas of shared experience and sense-making amongst our participants.

Jackie's account shows her days beginning and ending with a concern to monitor her medicine supplies, and the vignettes describe how these three participants have devised weekly temporal patterns of organising their DIY-dosette boxes to support daily routines. In Zac's case, trouser pockets stand in for boxes (he referred to his pockets as 'boxes' throughout one of our interviews). Zac's system allows him to adhere closely to taking his medicines at the prescribed times, which is a high priority for him. Maintaining his integrity as an active, helpful, independent, good citizen trumps any concerns he has for the integrity of the medicines in the storage conditions provided by his pockets. For Marco and Jackie, the preparation of the DIY-dosette boxes by Vicky and David, respectively, are important displays of reciprocity and part of a shared biography. In the case of Marco and Vicky, their mutual acts of organising form a tangible and important part of their ongoing support of each other.

In all cases, patients are balancing the perceived 'non-negotiable' requirement to take medicines with their desire for control over *how* they organise them into their lives. What emerges is inevitably a compromise. It is very difficult, perhaps impossible, for patients who are prescribed ten or more separate items of medication every day to take them strictly as prescribed, even when (as in our study) they express a clear intent to do so. As previous scholars have shown, there are practices of resistance,[35] and self-regulation,[36] within an overall context of reluctant acceptance of medicines as a way of life. All of our participants regard their organisation of their medicines as an expression of their desire to retain independence as far as this is possible, and to 'manage' themselves in a context of increasing dependency. Marco wants to avoid '*bothering*' his GP; Jackie does not

For peer review only - http://bmjopen.bmj.com/site/about/guidelines.xhtml

want to '*bother*' her neighbour about her eye drops. They all have strategies to ensure they do not run out of medicines.

Both Zac and Marco resist professional efforts to take over organising their medicines through the use of pharmacy-prepared MCCAs. They support this resistance with claims to cognitive capability. Marco's narrative points to the possibility of future decline when he may need to make use of the MCCA '*service*' but for now he constructs his identity as a capable, responsible user of both GP and pharmacy services who is prudent in ensuring he accumulates '*only a little surplus*' to avoid needless waste within a resource limited health system. Zac's persistent efforts to retain this position are ultimately thwarted.

DISCUSSION

Our study illuminates the organisational work that patients with multimorbidity do as they strive to adhere to their medicines in situations of complex polypharmacy. To our knowledge, no previous study has focused in detail on the work that goes on behind closed doors in the homes of older people prescribed ten or more items of medication. Polypharmacy requires organising, whether it occurs in the community pharmacy or at home. In both places this is demanding, time-consuming, and emotionally laden work. When doctors prescribe medicines, they prescribe work, but the nature, scale and complexity of this work usually remains invisible and unknown to the prescriber. The effort required by patients to do this work, and patients' capacity to do it are rarely discussed in formal processes of medication review. This work includes: practical acts of organising; surveillance of self and supplies; creative workarounds and experimentation with prescribed regimens; evaluation and management of personal priorities; anticipation and preparedness; management of self-presentation, itself a form of 'moral' work that includes the presentation of self as a 'good' adherent patient;[37] and securing the collaboration of carers and other professionals.

Previous research has identified households as 'hybrid centres of medication practice' where medicines are assimilated and many different forms of knowledge and expertise are brought together.[38] The key focus of our study is on individual patients, rather than households, but our findings show the extent to which individual

For peer review only - http://bmjopen.bmj.com/site/about/guidelines.xhtml

patients' practices are embedded in, and shaped by, social context and relational networks, and how patients build a particular kind of expertise in organising their medicines into their lives.

Our longitudinal study design has enabled us to locate polypharmacy within our participants' biographies and lived experiences. The 'ordinary' act of organising medicines emerges as a highly complex, deeply embodied and socially negotiated phenomenon. The patients who engage in this work typically regard it as unremarkable, mundane and taken-for-granted – a '*nuisance*'. The ethnographic gaze unpacks the scale and scope of this 'nuisance', makes visible the tacit knowledge underpinning the work, and brings into sharp focus the connectedness of medicines to meanings, social identities, practices and relationships. These connections extend far beyond the technical and biomedical concerns which are the typical focus of the clinic.

Our study is relatively small, involving 24 patients who use the services of three GP practices and four community pharmacies. We have prioritised depth of analysis over breadth which limits the generalisability of our findings, but has enabled a close focus on polypharmacy as 'work' that must be attended to, and offers clinicians insights into the consequences of their prescribing which are usually hidden from clinical view. Our participants were all committed to adhering as well as possible to their medicines regimens. We accept that this may not always be the case; our participants' willingness to take part in our research might be a reflection of their commitment to their medicines. However, we have shown that even this group of patients struggled to adhere to regimens exactly as prescribed and had to be very resourceful to meet the organisational demands of their treatment.

Pharmacy-prepared MCCAs or dosette boxes are often offered to patients as one way of supporting adherence in situations of polypharmacy; approximately 64 million are issued in England per year[39] although professional bodies discourage their use.[40 41] Royal Pharmaceutical Society (RPS) guidance clearly states: '*The use of original packs of medicines with appropriate support is the preferred option of supplying medicines in the absence of a specific need for an MCCA as an adherence intervention'.* Concerns include, but are not limited to: dispensing drugs in a MCCA is an unlicensed use of medicines; the limited evidence available (mostly

For peer review only - http://bmjopen.bmj.com/site/about/guidelines.xhtml

from care home settings) indicates a lack of patient benefit;[40] many medicines become unstable when removed from their original packaging (and drug manufacturers are not required to test the stability of medicines repackaged in a MCCA);[40] patients may lose confidence about their medicines; scope for waste when prescriptions change. Pharmacists have legal responsibility for assessing a patient's need for a MCCA and there have been recent calls to regulate pharmacists more heavily, to improve standards of MCCA practice and reduce the number issued.[39]

In this paper we have focused primarily on patients who are dispensed their medicines in their original packaging, as recommended in professional guidance (albeit Zac, like three other participants in our study went on to receive a MCCA part-way through our follow-up). Our observations highlight some of the parallels between the work of the pharmacy in preparation of MCCAs and the work that patients must undertake when they are responsible for medicines dispensed in large numbers in original packs. All of our participants devised ways of organising medicines that they could accommodate within their lives, often using DIY-MCCAs and often exposing their medicines to some of the same risks that underpin professional concerns about MCCAs (e.g. the risk of medicines losing their integrity when removed from their packaging). Some patients valued taking ownership of organising their own medicines and expressed reluctance at handing over this responsibility to pharmacists.

Calls to improve standards of MCCA practice are welcome.[39] However the goal of reducing their use in favour of dispensing medicines in their original packaging may fall short of the mark by failing to address the most complex issues at stake. Our work shows that the burden and risks of 'organising' polypharmacy in the service of medicines adherence always fall somewhere; the work does not go away. The most complex issue sustaining this work is polypharmacy itself. Even when robust 'evidence-based' arguments can be made for each individual item in a list of ten, fifteen or more drugs, the evidence supporting such extensive polypharmacy in older people with multimorbidities is questionable.[42] Combinations of drugs prescribed according to 'single disease' guidance (and often based on trials conducted in younger populations) can be both disruptive and dangerous in this context.[6 43] Unless the professional gaze shifts intentionally towards finding ways of tackling

For peer review only - http://bmjopen.bmj.com/site/about/guidelines.xhtml

polypharmacy itself, the ‘hidden’ burden of organising polypharmacy will always fall somewhere, and will always carry risk.

For peer review only

For peer review only - http://bmjopen.bmj.com/site/about/guidelines.xhtml

Funding statement

This article presents independent research funded by the National Institute for Health Research (NIHR) through a Clinician Scientist Award (DS). In addition NF was (in part) supported by the National Institute for Health Research (NIHR) Collaboration for Leadership in Applied Health Research and Care (CLAHRC) North Thames at Bart's Health NHS Trust. The views expressed are those of the author(s) and not necessarily those of the NHS, the NIHR or the Department of Health and Social Care.

Competing interests

None declared

Author contributions

DS designed the study and secured funding. DS and NF jointly gathered and analysed data. DS wrote the first draft of the paper. Both authors revised and finalised the manuscript.

Acknowledgements

We would like to thank the staff and patients who participated in this study. We would also like to thank members of our Expert Advisory Group and our patient panel for their valuable advice, and colleagues at QMUL who commented on an early draft of the manuscript.

Ethics Approval

The project has ethics approval from Leeds West Research Ethics Committee (IRAS project ID: 205517; REC reference 16/YH/0462).

For peer review only

Data sharing statement

Our ethics approval was granted based on the anonymity of the individuals consenting to participate and our participant information sheets and consent forms reflected this. Our research participants have not consented to making our raw data publicly available.

REFERENCES

1. World Health Organization. Medication Without Harm - Global Patient Safety Challenge on Medication Safety. Geneva: World Health Organization, 2017.
2. Guthrie B, Makubate B, Hernandez-Santiago V, et al. The rising tide of polypharmacy and drug-drug interactions: population database analysis 1995–2010. *BMC medicine* 2015;13(1):74.
3. Duerden M, Avery T, Payne R. Polypharmacy and medicines optimisation: Making it safe and sound. London: The King's Fund 2013.
4. Guthrie B, McCowan C, Davey P, et al. High risk prescribing in primary care patients particularly vulnerable to adverse drug events: cross sectional population database analysis in Scottish general practice. *BMJ* 2011;342:d3514.
5. Payne RA, Avery AJ. Polypharmacy: one of the greatest prescribing challenges in general practice. *British Journal of General Practice* 2011;61(583):83-84.
6. May C, Montori VM, Mair FS. We need minimally disruptive medicine. *BMJ* 2009;339:b2803.
7. McCoy L. Time, self and the medication day: a closer look at the everyday work of 'adherence'. *Sociology of health & illness* 2009;31(1):128-46.
8. National Institute for Health Care Excellence. Medicines optimisation: the safe and effective use of medicines to enable the best possible outcomes. Available from: https://www.nice.org.uk/guidance/ng52015.
9. Royal Pharmaceutical Society. Medicines Optimisation: Helping patients to make the most of medicines. Available at: http://www.rpharms.com/promoting-pharmacy-pdfs/helping-patients-make-the-most-of-their-medicines.pdf2013.
10. Cohen D, McCubbin M, Collin J, et al. Medications as social phenomena. *Health:* 2001;5(4):441-69.
11. NHS Improvement. Network contract directed enhanced service. Contract specification. England: NHS, 2020.
12. Conrad P. The meaning of medications: another look at compliance. *Social science & medicine* 1985;20(1):29-37.
13. Cohen MD, Bacdayan P. Organizational Routines Are Stored As Procedural Memory: Evidence from a Laboratory Study. *Organization Science* 1994;5(4):554-68.
14. Whyte SR, Van der Geest S, Hardon A. Social lives of medicines. Cambridge: Cambridge University Press 2002.
15. Webster A, Douglas C, Lewis G. Making sense of medicines:'lay pharmacology'and narratives of safety and efficacy. *Science as Culture* 2009;18(2):233-47.
16. Dew K, Norris P, Gabe J, et al. Moral discourses and pharmaceuticalised governance in households. *Social Science & Medicine* 2015;131:272-79.
17. Suchman L. Plans and situated actions: The problem of human-machine communication. Cambridge: Cambridge University Press 1987.
18. Kim J, Whitney A, Hayter S, et al. Development and initial testing of a computer-based patient decision aid to promote colorectal cancer screening for primary care practice. *BMC Medical Informatics and Decision Making* 2005;5(1):36.

For peer review only - http://bmjopen.bmj.com/site/about/guidelines.xhtml

19. Huyard C, Haak H, Derijks L, et al. When patients' invisible work becomes visible: non-adherence and the routine task of pill-taking. *Sociology of health & illness* 2019;41(1):5-19.
20. Dixon-Woods M. What can ethnography do for quality and safety in health care? *Quality and Safety in Health Care* 2003;12:326-27.
21. Hammersley M, Atkinson P. Ethnography: principles in practice. London: Routledge 1995.
22. Swinglehurst D, Fudge N. Addressing the polypharmacy challenge in older people with multimorbidity (APOLLO-MM): study protocol for an in-depth ethnographic case study in primary care. *BMJ open* 2019;9(8):e031601.
23. O'Brien BC, Harris IB, Beckman TJ, et al. Standards for reporting qualitative research: a synthesis of recommendations. *Academic Medicine* 2014;89(9):1245-51.
24. Fudge N, Swinglehurst D. 'It's all about patient safety': an ethnographic study of how pharmacy staff construct medicines safety in the context of polypharmacy. *BMJ open* 2021;11(2):e042504.
25. Wengraf T. Qualitative research interviewing : biographical narrative and semi-structured methods. London, Thousand Oaks, New Delhi: Sage Publications 2001.
26. Gaver B, Dunne T, Pacenti E. Design: cultural probes. *interactions* 1999;6(1):21-29.
27. Malterud K, Siersma VD, Guassora AD. Sample size in qualitative interview studies: guided by information power. *Qualitative health research* 2016;26(13):1753-60.
28. QSR International. NVivo Qualitative Data Analysis Software [program] Version 12 2018 [Available from: https://www.qsrinternational.com/nvivo-qualitative-data-analysis-software/home.
29. Schatzki TR. Introduction: practice theory. In: Schatzki TR, Knorr Cetina K, von Savigny E, eds. The Practice Turn in Contemporary Theory. Abingdon, New York: Routledge 2001:1-14.
30. Nicolini D. Practice Theory, Work, & Organization: An introduction. Oxford: Oxford University Press 2012.
31. Goffman E. Regions and Region Behaviour. The Presentation of Self in Everyday Life. London: Penguin Books 1959:109-40.
32. Palen L, Aaløkke S. Of pill boxes and piano benches: "home-made" methods for managing medication. *Proceedings of the 2006 20th anniversary conference on Computer supported cooperative work* 2006:79-88.
33. Mitchell JC. Producing data: Case studies. In: Ellen RF, ed. Ethnographic Research: A Guide to General Conduct. London: Academic Press 1984:237-41.
34. Geertz C. The Interpretation of Cultures. New York: Basic Books 1973.
35. Pound P, Britten N, Morgan M, et al. Resisting medicines: a synthesis of qualitative studies of medicine taking. *Social Science and Medicine* 2005;61:133-55.
36. Pollock K, Grime J. Strategies for reducing the prescribing of proton pump inhibitors (PPIs): patient self-regulation of treatment may be an under-exploited resource. *Social science & medicine* 2000;51(12):1827-39.
37. Hawking MK, Robson J, Taylor SJ, et al. Adherence and the Moral Construction of the Self: A Narrative Analysis of Anticoagulant Medication. *Qualitative health research* 2020;30(14):2316-30.
38. Dew K, Chamberlain K, Hodgetts D, et al. Home as hybrid centre of medication practice. *Sociology of Health and Illness* 2014;36:28-43.
39. Yeung A. North East and North Cumbria Medicines Adherence Support Project, 2019.
40. Royal Pharmaceutical Society. Improving patient outcomes–the better use of multi-compartment compliance aids: Royal Pharmaceutical Society Great Britain, 2013.
41. London Medicines Information Service. Summary of Guidance and Evidence for use of Multi-Compartment Compliance Aids, 2019.
42. Armstrong N, Swinglehurst D. Understanding medical overuse: the case of problematic polypharmacy and the potential of ethnography. *Family practice* 2018

For peer review only - http://bmjopen.bmj.com/site/about/guidelines.xhtml

For peer review only

43. Swinglehurst D, Hjörleifsson S. The everyday ethics of burdensome polypharmacy. *Public Policy & Aging Report* 2018;28(4):113-15.

For peer review only

For peer review only - http://bmjopen.bmj.com/site/about/guidelines.xhtml

Standards for Reporting Qualitative Research (SRQR)*

http://www.equator-network.org/reporting-guidelines/srqr/

Page/line no(s).

Title and abstract

**Title** - Concise description of the nature and topic of the study Identifying the study as qualitative or indicating the approach (e.g., ethnography, grounded theory) or data collection methods (e.g., interview, focus group) is recommended	Title page p1
**Abstract** - Summary of key elements of the study using the abstract format of the intended publication; typically includes background, purpose, methods, results, and conclusions	P2

Introduction

**Problem formulation** - Description and significance of the problem/phenomenon studied; review of relevant theory and empirical work; problem statement	Introduction P4-6
**Purpose or research question** - Purpose of the study and specific objectives or questions	P6, final paragraph of introduction

Methods

**Qualitative approach and research paradigm** - Qualitative approach (e.g., ethnography, grounded theory, case study, phenomenology, narrative research) and guiding theory if appropriate; identifying the research paradigm (e.g., postpositivist, constructivist/ interpretivist) is also recommended; rationale**	Ethnography briefly explained in Introduction (end of P5); practice theory explained in Analysis P8-9
**Researcher characteristics and reflexivity** - Researchers' characteristics that may influence the research, including personal attributes, qualifications/experience, relationship with participants, assumptions, and/or presuppositions; potential or actual interaction between researchers' characteristics and the research questions, approach, methods, results, and/or transferability	Sampling / data collection final paragraph p8
**Context** - Setting/site and salient contextual factors; rationale**	Setting P8
**Sampling strategy** - How and why research participants, documents, or events were selected; criteria for deciding when no further sampling was necessary (e.g., sampling saturation); rationale**	Data collection P8
**Ethical issues pertaining to human subjects** - Documentation of approval by an appropriate ethics review board and participant consent, or explanation for lack thereof; other confidentiality and data security issues	Ethics approval P 25
**Data collection methods** - Types of data collected; details of data collection procedures including (as appropriate) start and stop dates of data collection and analysis, iterative process, triangulation of sources/methods, and modification of procedures in response to evolving study findings; rationale**	Data collection Table 1 p.6 and reference to protocol paper

For peer review only - http://bmjopen.bmj.com/site/about/guidelines.xhtml

	for more detail (ref 22)
**Data collection instruments and technologies** - Description of instruments (e.g., interview guides, questionnaires) and devices (e.g., audio recorders) used for data collection; if/how the instrument(s) changed over the course of the study	Methods P 6-8
**Units of study** - Number and relevant characteristics of participants, documents, or events included in the study; level of participation (could be reported in results)	Table 1 P6 and description of 'case' (p8) and 'telling case' (p13)
**Data processing** - Methods for processing data prior to and during analysis, including transcription, data entry, data management and security, verification of data integrity, data coding, and anonymization/de-identification of excerpts	Analysis P 8 - 9
**Data analysis** - Process by which inferences, themes, etc., were identified and developed, including the researchers involved in data analysis; usually references a specific paradigm or approach; rationale**	
**Techniques to enhance trustworthiness** - Techniques to enhance trustworthiness and credibility of data analysis (e.g., member checking, audit trail, triangulation); rationale**	Analysis P 8 -9

Results/findings

**Synthesis and interpretation** - Main findings (e.g., interpretations, inferences, and themes); might include development of a theory or model, or integration with prior research or theory	Findings P9 – 21 including Tables 2-5
**Links to empirical data** - Evidence (e.g., quotes, field notes, text excerpts, photographs) to substantiate analytic findings	Findings P9 – 21 including Tables 2-5

Discussion

**Integration with prior work, implications, transferability, and contribution(s) to the field -** Short summary of main findings; explanation of how findings and conclusions connect to, support, elaborate on, or challenge conclusions of earlier scholarship; discussion of scope of application/generalizability; identification of unique contribution(s) to scholarship in a discipline or field	Discussion P21-24 and 'strengths and limitations' p 3
**Limitations** - Trustworthiness and limitations of findings	'strengths and limitations' p 3 Discussion para 2 P22

Other

**Conflicts of interest** - Potential sources of influence or perceived influence on study conduct and conclusions; how these were managed	P25
**Funding** - Sources of funding and other support; role of funders in data collection, interpretation, and reporting	P25

For peer review only - http://bmjopen.bmj.com/site/about/guidelines.xhtml

*The authors created the SRQR by searching the literature to identify guidelines, reporting standards, and critical appraisal criteria for qualitative research; reviewing the reference lists of retrieved sources; and contacting experts to gain feedback. The SRQR aims to improve the transparency of all aspects of qualitative research by providing clear standards for reporting qualitative research.

**The rationale should briefly discuss the justification for choosing that theory, approach, method, or technique rather than other options available, the assumptions and limitations implicit in those choices, and how those choices influence study conclusions and transferability. As appropriate, the rationale for several items might be discussed together.

Reference:

O'Brien BC, Harris IB, Beckman TJ, Reed DA, Cook DA. **Standards for reporting qualitative research: a synthesis of recommendations.** *Academic Medicine*, Vol. 89, No. 9 / Sept 2014 DOI: 10.1097/ACM.0000000000000388

For peer review only
